# Advances in the treatment of atherosclerosis with ligand‐modified nanocarriers

**DOI:** 10.1002/EXP.20230090

**Published:** 2023-12-07

**Authors:** Xiujiao Deng, Jinghao Wang, Shanshan Yu, Suiyi Tan, Tingting Yu, Qiaxin Xu, Nenghua Chen, Siqi Zhang, Ming‐Rong Zhang, Kuan Hu, Zeyu Xiao

**Affiliations:** ^1^ Department of Pharmacy The First Affiliated Hospital of Jinan University Guangzhou China; ^2^ The Guangzhou Key Laboratory of Basic and Translational Research on Chronic Diseases Jinan University Guangzhou China; ^3^ Provincial Key Laboratory of New Drug Screening, School of Pharmaceutical Sciences Southern Medical University Guangzhou China; ^4^ Department of Pharmacy Zhujiang Hospital Southern Medical University Guangzhou China; ^5^ State Key Laboratory of Bioactive Substance and Function of Natural Medicines, Institute of Materia Medica Chinese Academy of Medical Sciences and Peking Union Medical College Beijing China; ^6^ The Guangzhou Key Laboratory of Molecular and Functional Imaging for Clinical Translation Jinan University Guangzhou China; ^7^ Department of Advanced Nuclear Medicine Sciences, Institute of Quantum Medical, Science National Institutes for Quantum Science and Technology Chiba Japan

**Keywords:** atherosclerosis, drug delivery, ligand‐modified, nano‐drugs, targeted therapies

## Abstract

Atherosclerosis, a chronic disease associated with metabolism, poses a significant risk to human well‐being. Currently, existing treatments for atherosclerosis lack sufficient efficiency, while the utilization of surface‐modified nanoparticles holds the potential to deliver highly effective therapeutic outcomes. These nanoparticles can target and bind to specific receptors that are abnormally over‐expressed in atherosclerotic conditions. This paper reviews recent research (2018–present) advances in various ligand‐modified nanoparticle systems targeting atherosclerosis by specifically targeting signature molecules in the hope of precise treatment at the molecular level and concludes with a discussion of the challenges and prospects in this field. The intention of this review is to inspire novel concepts for the design and advancement of targeted nanomedicines tailored specifically for the treatment of atherosclerosis.

## Graphical abstract

Overview diagram of targeted ligand‐modified nanomedicines for the treatment of atherosclerosis. Ligand‐modified nanoparticles targeting specific atherosclerosis receptors at each pathological stage from monocyte recruitment, macrophage polarisation and foam cell formation, vascular smooth muscle cell phenotypic transformation, and vulnerable plaque formation, opening up ideas for the design of atherosclerosis‐targeted nanomedicines.

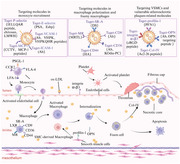

## 1 INTRODUCTION

Atherosclerosis is a major driver of cardiovascular disease (CVD) and a huge global burden of mortality and morbidity, with CVD‐related deaths expected to increase to approximately 23.6 million per year by 2030.^[^ ^1^ ^]^ Based on disturbances in arterial intima's lipid metabolism, atherosclerosis manifests as a chronic inflammatory disease.^[^ ^2^, ^3^ ^]^ The pathological progression of atherosclerosis encompasses endothelial injury, lipid accumulation, inflammatory cell infiltration, foam cell generation, and plaque advancement.^[^ ^4^ ^]^ The microenvironment within atherosclerotic plaques constitutes a multifaceted physical and biochemical milieu, comprising diverse cellular components and molecules, including endothelial cells (ECs), monocytes, macrophages, vascular smooth muscle cells (VSMCs), oxidized low‐density lipoprotein (ox‐LDL), inflammatory factors, chemokines, and other entities.^[^ ^5^, ^6^ ^]^

Currently, clinical treatment drugs mainly focus on lipid regulation, anti‐platelet aggregation, and inhibition of blood clot formation.^[^ ^7^ ^]^ These drugs can slow the progression of atherosclerosis, but they fail to specifically aggregate at plaque sites, do not specifically prevent plaque rupture, and may have systemic side effects.^[^ ^8^ ^]^ For example, statin‐associated muscle pain is common in clinical practice, limiting its long‐term use.^[^ ^9^ ^]^ Fortunately, the emergence of nano‐drug delivery technology has sparked fresh optimism in advancing the treatment of atherosclerosis.^[^ ^10^ ^]^ Nano‐drugs are pharmaceutical formulations or delivery systems that leverage nanotechnology to manipulate drug molecules or carriers at the nanoscale, typically less than 100 nm, aiming to achieve improved drug solubility, stability, targeted delivery, and controlled release.^[^ ^11^ ^]^ Because of its biocompatibility, biodegradability, and extended circulation time in the bloodstream, nano‐drug delivery is often selected as the preferred method for transporting a variety of therapeutic agents,^[^ ^12^ ^]^ including small molecule drugs, proteins, peptides, and even cellular therapies.^[^ ^13^ ^]^ Nanotherapeutic drugs can address the pharmacokinetic properties and stability in the chemical composition of most loaded therapeutic drugs and reduce systemic adverse effects.^[^ ^14^ ^]^

Due to the small size of plaques in atherosclerotic areas and the high intravascular blood flow, higher conditions are required for the effective enrichment of nanoparticles in plaques.^[^ ^6^ ^]^ Therefore, improved targeting of nano‐drugs is necessary for the treatment of atherosclerosis to maintain drug release along the route of administration and at the desired target site. Precise therapies at the molecular level can be facilitated by targeting essential molecules, including cell surface receptors, cytokines, and signalling pathway proteins.^[^ ^15^ ^]^ Surface‐specific modifications give nanomedicines the ability to target biomarkers over‐expressed in plaques, with positive enrichment at the site of the lesion, surmounting biological barriers and significantly enhancing the efficacy‐to‐toxicity ratio of the drug.^[^ ^16^ ^]^ Ligand‐modified nanocarriers refer to nanoparticles with specific ligand molecules attached to their surfaces. These ligands can include high‐affinity proteins, antibodies, peptides, small molecular compounds, and others capable of specific binding with target molecules, such as receptors or cell surface molecules, enabling targeted binding, recognition, and enhanced functionalities of the nanoparticles through specific interactions with target molecules. This review offers a fresh perspective on the design strategies of specifically targeted nanomedicines in atherosclerosis therapy (Table 1), while also discussing the existing challenges and promising prospects within this field. We hope that this study will inspire new ideas will be provided for the treatment of cardiovascular disease.

**TABLE 1:** Targeted nanomedicines for the treatment of atherosclerosis.

Carrier	Targeting molecules	Targeted ligands	Delivery agents	Cells in vitro	Animal model, administration route	Treatment effectiveness
Liposome^[^ ^35^ ^]^	VCAM‐1	Ab_VCAM‐1_	RelA siRNA	HUVEC		RelA expression↓, cell adhesion molecule expression↓
Micelle^[^ ^34^ ^]^	VCAM‐1	Ab_VCAM‐1_	GW0742	HAVSMCs		Cell apoptosis↓, cell migration↓
NP^[^ ^38^ ^]^	VCAM‐1	Ab_VCAM‐1_	EPA:DHA 6:1	mECs		Upregulation of VCAM‐1↓, upregulation of p53↓
NP^[^ ^45^ ^]^	VCAM‐1	CVHPKQHR	RAP	HUVEC, RAW264.7	ApoE^−/−^mice fed with HFD for 10 weeks, IV	The size of plaque lesions↓, the formation of intravascular plaque↓
Micelle^[^ ^41^ ^]^	VCAM‐1	VHPKQHR	microRNA‐92a	HAECs	ApoE^−/−^mice on PCL followed by HFD, IV	Aortic root AS↓
Liposome^[^ ^44^ ^]^	VCAM‐1	VHPKQHR	rapamycin	MAECs	ApoE^−/−^ mice aged 8 weeks and fed HFD for 4 months, IV	Aortic fluorescence intensity↓, T2 relaxation time↑
NP^[^ ^42^ ^]^	VCAM‐1	VHPK peptides	Anti‐microRNA‐712	iMAECs	Using PCL in C57BL/6 mice, IV	miR‐712 expression↓, TIMP3 expression↑
NP^[^ ^43^ ^]^	VCAM‐1	VHPK peptides	IL‐10	mECs and HUVEC	ApoE^−/−^ and Ldlr^−/−^ mice were fed HFD for 9∼21 weeks, IV	VCAM‐1 expression↓, IL‐10 production↑, inflammation within the plaque↓
Liposome^[^ ^53^ ^]^	ICAM‐1	Ab_ICAM‐1_	Pioglitazone		Yucatan minipigs undergoing hyperlipidaemic diet and balloon stripping, IV	AS formation↓
NP^[^ ^58^ ^]^	E‐selectin	Polysialic acid	Budesonide, l‐arginine	HUVEC	ApoE^−/−^ mice and IRI mice, IV	AS plaque load↓, vasodilation↑
NP^[^ ^62^ ^]^	E‐selectin	Esbp	dexamethasone	Spleen cells	ApoE^−/−^ mice were fed HFD for 8 weeks, IV	AS plaques↓, wall thickening of the ascending aortas↓
Liposome^[^ ^61^ ^]^	E‐selectin	Esbp	ATO, Curcumin	HAECs	ApoE^−/−^ mice aged 8 weeks with HFD for 8 weeks, IV	Reduced foam cell formation↓, IL‐6↓, MCP‐1↓
Liposome^[^ ^70^ ^]^	P‐selectin	P‐selectin peptide	siRNA	b.End3 cells		Specific silencing of siRNA
NP^[^ ^68^ ^]^	P‐selectin	chitosan		RAW 264.7, MVECs	ApoE^−/−^ mice with WD for 12 weeks, IV	Aortic root lesions↓, plaque area↓
Liposome^[^ ^71^ ^]^	P‐selectin	P‐selectin peptide	RAGE‐short hairpin RNA	bEnd.3 cells and THP‐1 cells	Male ApoE^−/−^ mice with HFD, IV	NF‐κB↓, TNF‐α↓, and RAGE protein expression in the aorta↓
Micelle^[^ ^67^ ^]^	P‐selectin	LMWH	indomethacin	RAW264.7; HUVECs	ApoE^−/−^ mice with HFD for 4 weeks, IV	ROS↓, TNF‐α↓, MMP‐2/9↓
NP^[^ ^78^ ^]^	MCP‐1	4‐mer peptide		RAW 264.7, J774 cells, BMDM	Male ApoE^−/−^ mice aged 5 weeks with HFD for at least 20 weeks, IV	inflammation↓
Micelle^[^ ^79^ ^]^	MCP‐1	MCP‐1 peptides	Col‐1 peptides	WEHI 1.1Mouse monocytes	ApoE^−/−^ mice aged 24 weeks with WD for 6 weeks, IV	plaque stability↑
NP^[^ ^92^ ^]^	SR‐A	dextran sulfate		RAW 264.7	Male ApoE^−/−^ mice HFD for 8∼12 weeks, IV	↓T2 signal in the wall of the ascending aortic plaques
NP^[^ ^93^ ^]^	SR‐A	dextran sulfate	Chlorin e6	RAW 264.7	ApoE^−/−^ mice (6∼8 weeks old, male) with HFD, IV	Pro‐inflammatory cytokines↓, carotid artery wall area↓
NP^[^ ^96^ ^]^	CD9	Ab_CD9_	rosuvastatin	RAW264.7, MS1 cells	ApoE^−/−^ mice aged 9 weeks fed HFD for 5 weeks, IV	Reactive oxygen species levels↓, TNF‐α↑, IL‐6↑, AS progression↓
NP^[^ ^107^ ^]^	CD36	KOdia‐PC	RAP	RAW 264.7		Rapid internalization by macrophages
NP^[^ ^106^ ^]^	CD36	KOdia‐PC		THP‐1 derived macrophages	Male LDLr^−/−^ mice aged 6 weeks fed with WD for 22 weeks, IV	Accumulate in areas of aortic lesions↑
NP^[^ ^103^ ^]^	CD36	KOdia‐PC	Epigallocatechin gallate	Mouse peritoneal macrophages	Male LDLr^−/−^ mice aged 6 weeks fed with WD for 22 weeks, IV	MCP‐1↓, TNF‐α↓, IL‐6 ↓
NP^[^ ^101^ ^]^	CD36	Ab_CD36_	SRT1720	RAW264.7	Male ApoE^−/−^ mice aged 6 weeks with HFD for 12 weeks, Abdominal injection	Total serum cholesterol↑, aortic plaque status ↓
NP^[^ ^113^ ^]^	CD44	HA	LOX‐1 siRNA, ATO	HUVEC, Human THP‐1 monocytes	Male ApoE^−/−^ mice aged 5 weeks with HFD for 16 weeks, IV	Plaque size↓, lipid accumulation↓, cytokine secretion↓
NP^[^ ^112^ ^]^	CD44	HA	ATO	RAW264.7	ApoE^−/−^ mice aged 20 weeks with HFD for 6 weeks, IV	AS plaque inflammation↓
NP^[^ ^117^ ^]^	CD44	HA	heparin	RAW 264.7 and HUVEC	New Zealand White rabbit exposed to FeCl_3_ on the isolated common carotid artery, Ear Vein Injection	Controlled drug release and chemotherapeutic synergy
NP^[^ ^111^ ^]^	CD44	HA	SIM	RAW 264.7 and HUVEC	ApoE^−/−^ mice with HFD for 10 weeks, IV	AS plaques↓, retaining the intact lumen area to maintain excellent blood flow
Liposome^[^ ^114^ ^]^	CD44	HA	SIM	MAECs and RAW 264.7	ApoE^−/−^ mice aged 5 weeks with HFD for 16 weeks, IV	Plaque size↓, lipid deposition↓, local inflammatory factor levels↓
NP^[^ ^121^ ^]^	CD44	HA		RAW264.7 and HUVEC	Male ApoE^–/–^ mice aged 6∼8 weeks on PLCA, IV	TNF‐α↓, IL‐6↓, macrophages↓, inflammation↓
NP^[^ ^116^ ^]^	CD44	HA	Rosuvastatin	RAW264.7, J774A.1, and THP‐1 cells	ApoE^−/−^ mice by implanting a cannula in the left carotid artery, followed by HFD for 12 weeks, IV	Plaque area↓, volume↓
Micelle^[^ ^115^ ^]^	CD44	HA	SIM	RAW264.7 and LO2 cells	ApoE^−/−^ mice aged 6 weeks with HFD for 8 weeks, IV	Cholesterol plaque levels↓
NP^[^ ^124^ ^]^	MR	MRTL		Rat's peritoneal macrophage		High uptake in M2 macrophages
NP^[^ ^142^ ^]^	profilin‐1	Ab_profilin‐1_	RAP	MOVAS	Male ApoE^−/−^ mice aged 12 weeks with HFD for 16 weeks, intraperitoneal injection	Alleviated the progression of arteriosclerosis
NP^[^ ^148^ ^]^	OPN	Ab_OPN_	PPARδ agonist GW1516	MOVAS	ApoE^−/−^ mice with HFD for 16 weeks, IV	Diminish the development of atherosclerosis
NP^[^ ^150^ ^]^	OPN	OPN targeting peptide	tirapazamine	RAW 264.7	Male ApoE^−/−^ mice aged 7 weeks were placed in a polymethyl pentene cast around the left common carotid artery and fed with HFD for 16 weeks, IV	Plaque area↓, carotid stenosis↓
NP^[^ ^149^ ^]^	OPN	OPN targeting peptide	SRT1720	MOVAS	Male ApoE^−/−^ mice aged 8 weeks with HFD for 12 weeks, IV	Necrotic core↓, collagen content↑
NP^[^ ^153^ ^]^	Integrin αvβ3	cRGD peptide	IL‐10	RAW 264.7	Female ApoE^−/−^ mice aged 6 weeks with WD for 10 weeks, IV	IL‐1β↓ plaque size↓
Liposome^[^ ^154^ ^]^	Integrin αvβ3	cRGD peptide	IL‐10	RAW 264.7		IL‐1β↓, TNF‐α↓
NP^[^ ^155^ ^]^	Integrin αvβ3	c(RGDfC)	RAP	HUVECs, HASMCs, RAW264.7	ApoE^−/−^ mice aged 6 weeks with HFD for 8 weeks, IV	Expression of MMP‐9 in aortic root plaques↓
NP^[^ ^163^ ^]^	Col IV	Peptide Ac2‐26	LXR agonist GW3965	Peritoneal macrophages	Ldlr^−/−^mice fed with WD for 14 weeks	LXR target genes↑, pro‐inflammatory mediators in macrophages↓
NP^[^ ^169^ ^]^	CD47 and integrin α4/β1	Ab_CD47_	Colchicine	RAW 264.7, HUVECs	Male ApoE^−/−^ mice aged 8 weeks with HFD and underwent PLCA, IV	Development of the atherosclerotic plaque↓
NP^[^ ^165^ ^]^	VCAM‐1 and CD44	Oxidized dextran	Astaxanthin and SS‐31 peptide	RAW 264.7	ApoE^−/−^ mice fed with HFD for 13 weeks, IV	Expression of ABCA1/G1 protein↑, LOX‐1/CD36 expression↓
NP^[^ ^164^ ^]^	VCAM‐1 and CD44	Dextran	Prednisolone and lipid‐specific AIEgen	RAW 264.7, HUVECs	ApoE^−/−^ mice with HFD for 4 weeks, IV	Actively enriched in AS
NP^[^ ^170^ ^]^	VCAM‐1, ICAM‐1, P‐selectin	Ab_VCAM‐1_, Ab_ICAM‐1_, sLex polymers		bEnd.3 cells	ApoE^−/−^ mice aged 5–6 weeks with HFD for 8 weeks, IV	Concentrations of TNF‐α↑, signal enhancement properties for ultrasound molecular imaging
Micelle^[^ ^166^ ^]^	E‐selectin and CD44	*N*‐acetylneuraminic acid, Chondroitin sulphate	RAP	HUVECs and Raw264.7	ApoE^−/−^ mice with HFD for 3 months, IV	Specific fluorescent substances gather at the aorta, ROS level↓
NP^[^ ^168^ ^]^	SR‐A and integrin αIIbβ3	Peptides PP1 and cyclic RGD	Fe3O4 and perfluoropentane	RAW 264.7	Male ApoE^−/−^ mice aged 6 weeks with WD for 16 weeks, IV	Precise targeting and therapeutic impact via anti‐inflammatory and thrombolytic properties
NP^[^ ^167^ ^]^	SR‐B1 and CD36	CD36 ligand and phosphatidylserine	Pitavastatin and SR‐A siRNA	RAW264.7	ApoE^−/−^ mice with HFD for 16 weeks, IV	65.8% reduction in plaque area and a 57.3% reduction in macrophages

Abbreviations: Ab, antibody; AS, atherosclerosis; ATO, atorvastatin; Col IV,Collagen IV; FLCs, Macrophage‐derived foam‐like cells;HA, hyaluronic acid; HAECs, Human aortic endothelial cells; HASMCs, human arterial smooth muscle cells; HAVSMCs, human aortic vascular smooth muscle cells; HFD, high‐fat diet; HUVEC, human umbilical vein endothelial cells; iMAECs, Immortalized mouse aortic endothelial cells; IV, Intravenous Injections; LMWH, low molecular weight heparin; MAECs, Murine aortic endothelial cells; mECs, Primary mouse endothelial cells; MMPs, matrix metalloproteinases; MOVAS, mouse aortic smooth muscle cells; MPMs, mouse peritoneal macrophages; MPPM, mouse peritoneal primary macrophages; MVECs, mouse vascular endothelial cells; NP, nanoparticle; OPN, Osteoblastin.;PAD4, protein arginine deiminase‐4; PCAL, partial carotid artery ligations; PCL, partial carotid ligation; PLCA, partial left carotid artery ligation; RAP, rapamycin; ROS, Reactive Oxygen Species; SIM, simvastatin; VECs, vascular endothelial cells; WD, Western diet.

## 2 TARGETING MOLECULES IN MONOCYTE RECRUITMENT

The initial step in the advancement of atherosclerotic plaques involves the recruitment of monocytes to the arterial wall.^[^ ^17^ ^]^ It is thought to follow the general paradigm of leukocyte adhesion and transport, including rolling, adhesion, and migration.^[^ ^18^ ^]^ The expression of lymphocyte function‐associated antigen 1 (LFA‐1), L‐selectin, p‐selectin glycoprotein ligand 1 (PSGL‐1), and very late integrin antigen 4 (VLA‐4) contribute to leukocyte adhesion and migration.^[^ ^19^ ^]^ At this time, the expression of intercellular adhesion molecule 1 (ICAM‐1), vascular cell adhesion molecule 1 (VCAM‐1), E‐selectin, and P‐selectin was increased in endothelial cells.^[^ ^20^ ^]^ They can bind coupled to receptors on monocytes. VCAM‐1 and ICAM‐1 interact with VLA‐4 and LFA‐1 on monocytes, respectively, to cause monocytes in the blood to adhere and translocate into the vessel wall.^[^ ^21^ ^]^ Simultaneously, the engagement of PSGL‐1 on monocytes with P‐selectin and E‐selectin promotes the capture and rolling of monocytes along the endothelial surface.^[^ ^22^ ^]^ Moreover, the presence of inflamed endothelium prompts activated ECs to release pro‐inflammatory chemokines, including monocyte chemotactic protein‐1(MCP‐1).^[^ ^23^ ^]^ These chemokines facilitate the translocation of monocytes to the subendothelial space by binding to C─C chemokine receptor 2 (CCR2) signals expressed on monocyte surfaces.^[^ ^24^ ^]^

Targeting the inhibition of monocyte recruitment is regarded as a promising therapeutic objective due to its potential to prevent the subsequent accumulation and proliferation of macrophages within the plaque.^[^ ^25^ ^]^ Additionally, it can aid in averting the destabilization and rupture of the atherosclerotic plaque.^[^ ^26^ ^]^ The administration of antibodies blocking VCAM‐1 or ICAM‐1 inhibits inflammatory monocyte recruitment during acute inflammation.^[^ ^27^ ^]^ Therefore, targeting the molecules VCAM‐1, ICAM‐1, P‐selectin, E‐selectin, and MCP‐1 during the monocyte recruitment process can be utilized to enhance the targeting of anti‐atherogenic drugs to maintain drug release along the route of administration and at the desired target site.

### 2.1 VCAM‐1 specific targets

VCAM‐1, a transmembrane protein classified under the immunoglobulin superfamily, serves as an adhesion molecule expressed on the surface of endothelial cells that have been activated.^[^ ^28^, ^29^ ^]^ Due to its strong affinity and interaction with leukocytes, VCAM‐1 serves as a valuable biomarker molecule for aberrant endothelial cell targeting, facilitating the recruitment of monocytes to endothelial cells.^[^ ^28^ ^]^ During the early and progressive stages of plaque formation, the presence of VCAM‐1 in atherosclerotic lesions remains a contributing factor to the pathological advancement of the disease.^[^ ^30^ ^]^ The glycoprotein α4β1 integrin, highly expressed on leukocyte membranes, is used for specific recognition and binding to VCAM‐1.^[^ ^31^ ^]^ Among the plethora of biomarkers associated with inflamed endothelium, VCAM‐1 has emerged as one of the extensively studied target sites for endothelial activation.^[^ ^32^ ^]^ Several drug delivery systems targeting VCAM‐1 have been developed to facilitate the delivery of drugs specifically to inflamed endothelial cells.

Researchers found that VCAM‐1 antibody loading on the surface of nanoparticles can improve the targeting ability of nanomedical drugs. The specific binding between VCAM‐1 antibody and VCAM‐1 involves the complementary interaction of the antibody's binding site with a specific region on the VCAM‐1 molecule's surface, forming a stable complex through non‐covalent forces such as hydrogen bonding, ion interactions, and spatial fitting.^[^ ^33^ ^]^ VCAM‐1 antibody can bind to the maleimide groups on the surface of nanocarriers such as nano micelles, liposomes, and nanoemulsions to overcome the application limitations of hydrophobic drugs^[^ ^34^ ^]^ and siRNA^[^ ^35^ ^]^ and improve the targeted accumulation of nanocarriers. Wei et al., by using the properties of high hydrophilicity and good biocompatibility of nanomicelles,^[^ ^36^ ^]^ conjured anti‐VCAM‐1 antibodies to the nanoparticles encapsulated with the hydrophobic drug GW0742 (PPARδ agonist) exhibited inhibition of apoptosis and migration in ox‐LDL‐induced HAVSMCs.^[^ ^34^ ^]^ He et al. formulated liposomes, called MOS and MSS, encapsulating RelA siRNA and coupling anti‐VCAM‐1 antibody targeting on the surface to increase the association with activated ECs.^[^ ^35^ ^]^ Negative‐charged siRNA can bind to positively‐charged liposomes through electrostatic interactions, forming stable complexes, and surface‐modified antibodies can enable targeted liposome delivery for enhanced release in specific cells or tissues. Of concern, increasing the number of cationic lipids in liposomes or using more portable headgroup cationic lipids (such as tertiary amines and pyridine) formulations improved siRNA transfection efficiency but also increased non‐specific associations with ECs.^[^ ^37^ ^]^ Fortunately, VCAM‐1 antibody coupling to the surface of liposomes minimizes differences in cationic lipid‐dependent cell associations. Additionally, Belcastro et al. found that the reduction of the size of VCAM‐1 antibody‐modified nano‐carriers is conducive to improving the targeting efficiency.^[^ ^38^ ^]^ They believed that the smaller the particles, the higher the particle concentration and the surface area exposed to specific ligands, which is conducive to targeting. In addition, smaller particles diffuse more quickly, facilitating faster ligand/receptor interactions. Unfortunately, the study of VCAM‐1 antibody‐modified nanocarriers has seen more results in vitro experiments, and its efficacy needs to be further studied in vivo.

Peptide ligands are emerging as a promising alternative to antibodies because of several advantages, including reduced molecular weight, convenient handling, and storage, evasion of the reticuloendothelial system, and strong specificity and affinity for the desired target.^[^ ^39^ ^]^ The VHPK peptide consists of the amino acids Val‐His‐Pro‐Lys and is widely used to target the VCAM‐1 due to its high affinity.^[^ ^40^ ^]^ As reported here, multiple VCAM‐1 targeting strategies have been explored for nanoparticle‐mediated targeted delivery of RNA,^[^ ^41^, ^42^ ^]^ DNA,^[^ ^43^ ^]^ and small molecule drugs.^[^ ^44^, ^45^ ^]^

Delivery of therapeutic nucleotides to target diseased tissues in vivo is usually not easy because nucleic acids are small, have a small charge, and are unstable.^[^ ^46^ ^]^ Polyelectrolyte complex micelles demonstrate remarkable potential as gene delivery carriers, as they can encapsulate charged nucleotides to establish a core through self‐assembly, neutralizing charges.^[^ ^41^ ^]^ This process not only safeguards nucleic acids against nonspecific interactions and enzymatic degradation but also contributes to their protective function. In addition, Poly(β‐amino ester) nanoparticles (pBAE NPs) are also promising vectors for nucleic acid delivery due to their extremely low toxicity, excellent biocompatibility, and rapid degradation kinetics.^[^ ^47^ ^]^ In particular, pBAE NPs allow RNA therapies to be fixed in polymer matrices, resulting in higher encapsulation and RNAi loading efficiency.^[^ ^48^ ^]^ Dosta et al. engineered a pBAE NP as a targeted delivery system for anti‐microRNA‐712 to specifically target inflammatory endothelial cells.^[^ ^42^ ^]^ Similarly, Distasio et al. used VHPK peptides electrostatically bound to pBAE NPs for the transportation of IL‐10 plasmid DNA, an anti‐inflammatory gene, and investigated the safety, biodistribution, and targeting potential of the NP formulations.^[^ ^43^ ^]^ To improve the targeting ability of NPs, the authors used a bifunctional hydrophilic coating polymer (pHPMA‐TT) containing a 2‐thiazoline‐2‐thiol group (TT group) that reacts with primary amines. 40% of the TT group was pre‐modified with *N*‐(2‐aminoethyl) maleimide, which allowed for the attachment of VHPK targeting peptides to terminal cysteine amino acids via a maleimide‐thiol reaction. The coupling both shielded the pBAE cationic charge and provided the pBAE NPs with atherosclerotic targeting ability.

There is substantial evidence that targeting peptides linked or encapsulated by liposomes can be used to target or encapsulate rapamycin (Rap) for disease therapy. The use of large doses of Rap can cause serious side effects such as dyslipidemia in vivo, so reducing the dosage of Rap is a necessary design strategy to achieve therapeutic effect.^[^ ^44^ ^]^ Chen et al. integrated ultramicro paramagnetic iron oxide (USPIO/Fe3O4) and Rap to create Rap/Fe3O4@VHP‐Lipo, illustrating the diagnostic and therapeutic advantages for early‐stage atherosclerosis.^[^ ^44^ ^]^ They used a lower Rap dose to achieve the same therapeutic effect as a conventional dose, showing the promising application of this targeted nanoparticle. Cell membrane mimetic design strategies combined with targeting VCAM‐1 have also been investigated for atherosclerosis treatment. Zhong et al. designed an erythrocyte membrane‐encapsulated and rapamycin‐loaded poly(lactic acid‐hydroxyacetic acid) (PLGA) nanocarrier to target atherosclerosis.^[^ ^45^ ^]^ Erythrocyte membrane vectors significantly extend blood circulation time, which is critical for effective in vivo drug delivery. After integrating the target function of the VCAM‐1 peptide‐modified nanoplatform, the accumulation within plaque was significantly increased, and its anti‐atherosclerotic potential was increased. In general, the coupling of VCAM‐1 targeting peptides has been investigated as an effective and promising targeting strategy, but its safety needs to be further validated in future large animal studies and human clinical trials.

### 2.2 ICAM‐1 specific targets

ICAM‐1, an immunoglobulin superfamily member, is a cell surface glycoprotein. While endothelial cells express it at low basal levels, its expression is upregulated in response to inflammatory stimuli.^[^ ^49^ ^]^ Furthermore, ICAM‐1 linkage to monocytes has been shown to trigger trans‐endothelial migrations of monocytes under conditions that mimic inflammation.^[^ ^50^ ^]^ Thus, the upregulation of ICAM‐1 serves as a pivotal factor in driving the inflammatory response.

Targeted ICAM‐1 molecule based on ICAM‐1 antibody coupling has been investigated for use in both diagnosing and treating atherosclerosis.^[^ ^51^, ^52^, ^53^ ^]^ Li et al. used ICAM‐1 antibody to prepare ICAM‐1‐targeted ultrasound nanobubbles for the dynamic observation of different stages of atherosclerotic inflammatory lesions in rabbits, which were shown to accurately reflect the extent of inflammatory damage at various stages of atherosclerotic lesions and were validated by pathological findings.^[^ ^51^ ^]^ However, the binding rate of ICAM‐1 antibody to nanobubbles in this study is not high, and more suitable synthesis methods need to be found to improve the binding rate. Bertrand et al. used a labelled imaging tracer probe that combined an ICAM‐1 antibody and a collagen‐binding peptide for near‐infrared fluorescence imaging after local delivery at the site of abdominal aortic injury in rabbits.^[^ ^52^ ^]^ Of interest, Kee et al. infused anti‐ICAM‐1‐coupled echo‐liposomes (ELIP) loaded with pioglitazone directly into the artery and used ultrasound to facilitate the entry of the preparation into the peripheral artery of the stent to achieve a sustained anti‐inflammatory effect.^[^ ^53^ ^]^ The use of ICAM‐1 antibody‐coupled nanoparticles for short‐term imaging is limited; further discussion is needed to design nanomedical drugs for anti‐atherosclerosis therapy.

### 2.3 E‐selectin specific targets

Following inflammatory stimuli, E‐selectin, an adhesion molecule, undergoes significant upregulation in activated ECs,^[^ ^54^ ^]^ and is effectively internalized upon ligand binding, making it an ideal target epitope.^[^ ^55^ ^]^

**FIGURE 1:**
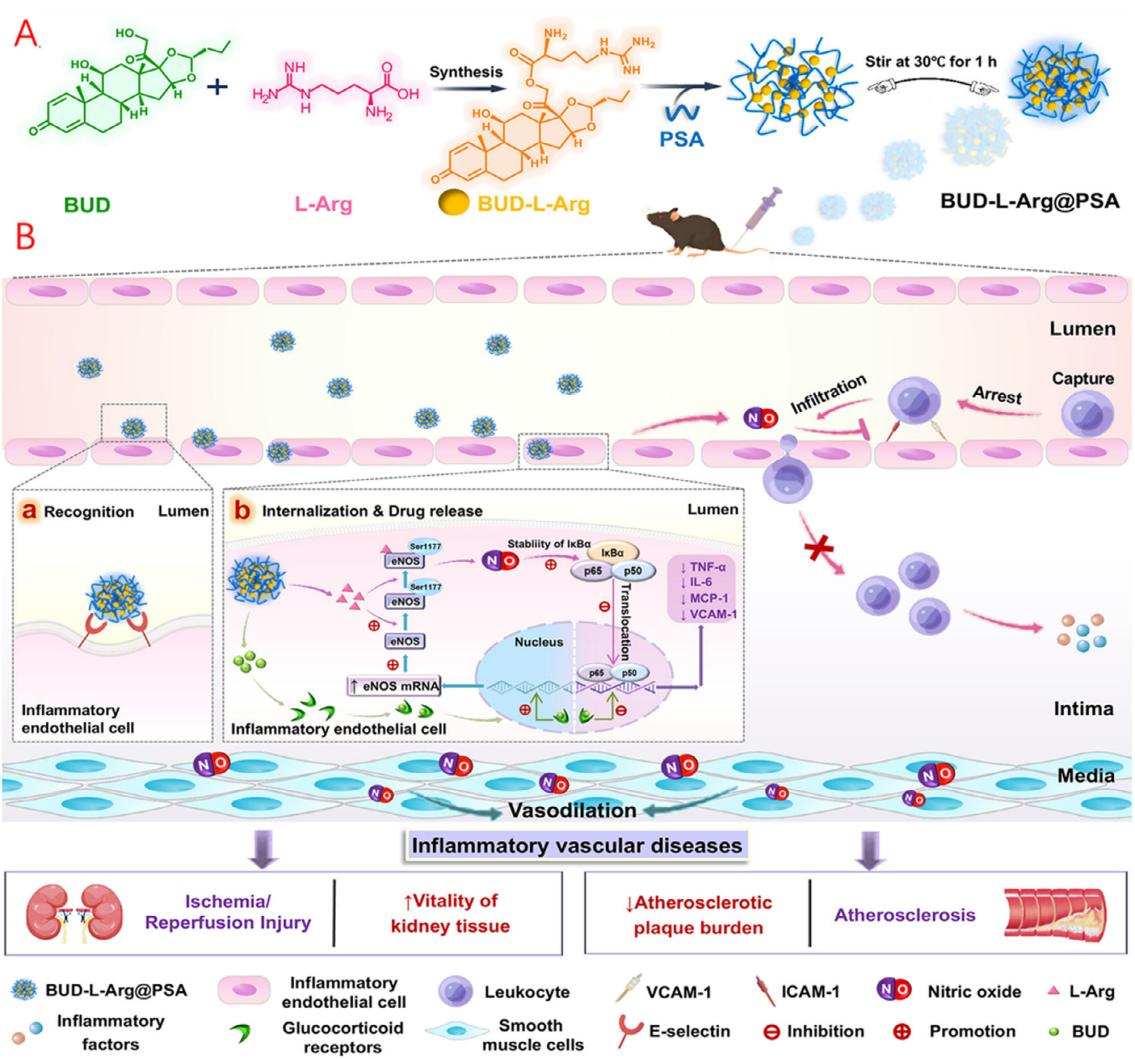
Nanopolymers BUD‐L‐Arg@PSA for targeted treatment of atherosclerosis. (A) Schematic diagram of the preparation of the nanopolymers. (B) Targeted delivery and therapeutic mechanisms of BUD and L‐Arg via PSA affinity for E‐selectin. Adapted with permission.^[^ ^58^ ^]^ Copyright 2023, American Chemical Society.

Polysialic acid (PSA) is a naturally occurring hydrophilic polysaccharide characterized by low viscosity and non‐immunogenic properties, undergoing in vivo degradation.^[^ ^56^ ^]^ PSA contains an α−2,8‐ketoglycoside‐linked sialic acid monomer that binds to E‐selectin.^[^ ^57^ ^]^ By capitalizing on the unique binding affinity between PSA and E‐selectin, Wang et al. successfully encapsulated PSA with budesonide (BUD) and l‐arginine (l‐Arg) to form nanopolymers (BUD‐l‐Arg@PSA).^[^ ^58^ ^]^ The researchers achieved this by chemically merging BUD and l‐Arg through an ester bond, resulting in the creation of a dual precursor drug known as BUD‐l‐Arg (as shown in Figure 1A). Subsequently, under stirring conditions, PSA and BUD‐l‐Arg were co‐loaded into nanopolymers using various intermolecular non‐covalent interactions. It is noteworthy that the oxidation of l‐Arg generates NO,^[^ ^59^ ^]^ and the combined anti‐inflammatory properties of BUD complementarily inhibit the production of inflammatory factors within the NF‐κB signalling pathway (Figure 1B). In a mouse atherosclerosis model, these nanopolymers alleviate atherosclerotic plaques and improve vasodilation.

An alternative targeting strategy is the use of high‐affinity E‐selectin binding peptides (Esbp). Esbp contains the sequence DITWDQLWDLMK, which is found and binds E‐selectin but not its family members P‐selectin and L‐selectin.^[^ ^60^ ^]^ Liu et al. prepared liposomes (T‐AC‐Lipo) using Esbp modification to specifically bind E‐selectin to deliver both atorvastatin calcium (Ato) and curcumin (Cur) to dysfunctional ECs.^[^ ^61^ ^]^ Through targeted liposomal delivery, Ato and Cur were transported to malfunctioning ECs, leading to a cooperative inhibition of adhesion molecules (E‐selectin and ICAM‐1) and plasma lipid levels. Differently, Tsoref et al. utilized *N*‐(2‐hydroxypropyl)methacrylamide (HPMA) copolymers with Esbp as targeting ligands to bind E‐selectin for targeting functions.^[^ ^62^ ^]^ The combination of dexamethasone (Dex) is named P‐(Esbp)‐Dex and without Dex is P‐Esbp. The study demonstrated that both of them effectively decreased wall thickening in the ascending aorta following a 2‐week treatment period. Notably, treatment with P‐Esbp resulted in a significant reduction in the size of necrotic cores within plaques and effectively shifted splenic macrophages towards an anti‐inflammatory phenotype. They created a drug‐free macromolecular therapy to treat vascular inflammation. The therapy uses a water‐soluble nano‐biomedical polymer containing a multicopy Esbp that efficiently targets aortic plaque when injected intraperitoneally.

### 2.4 P‐selectin specific targets

Belonging to the selectin family of cell adhesion molecules, P‐selectin is highly expressed in inflammatory endothelium, increasing platelet‐leukocyte aggregation and leukocyte‐endothelial adhesion.^[^ ^63^ ^]^ P‐selectin binds to monocytes via PSGL‐1,^[^ ^64^ ^]^ which enhances NF‐κB nuclear translocation and release of TNF‐α.^[^ ^65^ ^]^ P‐selectin/PSGL‐1 interaction provides a promising therapeutic target for addressing inflammatory diseases.^[^ ^66^ ^]^ Various forms of nanoparticles have been surface‐covered with ligands (e.g. sulphated oligosaccharides,^[^ ^67^, ^68^ ^]^ peptides^[^ ^69^, ^70^, ^71^ ^]^ etc.) that bind to the highly expressed P‐selectin on the surface of activated ECs.

Previous studies have demonstrated that sulphated oligosaccharides (e.g. rockrose dextran, dextran sulphate, and heparin) can effectively target P‐selectin, which is positively correlated with metastatic growth.^[^ ^72^ ^]^ Wang et al. demonstrated that low molecular weight heparin (LMWH) competitively binds P‐selectin.^[^ ^67^ ^]^ LMWH, as a polysaccharide molecule, possesses numerous negatively charged sulphate groups, which can interact with specific domains on P‐selectin.^[^ ^73^ ^]^ The authors designed and synthesized an amphiphilic precursor coupling LMWH to indomethacin (IND), which has revealed the capacity for self‐assembly into stable and controlled‐release precursor micelles (LI NPs) that are biocompatible. LMWH and IND are two agents that can help prevent vascular inflammation and plaque development. LMWH works by binding competitively to P‐selectin, which helps to stop monocytes and macrophages from being recruited by ECs. This effectively obstructs the initial phase of vascular inflammation. IND, on the other hand, reduces the level of reactive oxygen species (ROS) and inhibits the secretion of pro‐inflammatory factors by plaque macrophages. This helps to prevent the advancement of plaque development. Fucoidan, a sulphated polysaccharide sourced from marine organisms, is a promising candidate for nanoparticles aimed at atherosclerosis treatment due to its specific targeting ligand for P‐selectin and numerous favourable biological properties. Fucoidan's structure features an α(1‐3)‐l‐fucose linear backbone with sulphate substitution, facilitating its high‐affinity binding to P‐selectin.^[^ ^74^ ^]^ Consequently, the exploration of nanoparticle formulations rooted in fucoidan and specific to P‐selectin represents a significant avenue in atherosclerosis therapy. Jafari et al. developed Fucoidan–Chitosan Nanoparticles (CFN)^[^ ^68^ ^]^ by exploiting the high‐affinity binding of chitosan to P‐selectin.^[^ ^74^ ^]^ In vivo experiments have shown that they can effectively inhibit local oxidative stress and inflammation, thereby stopping the progression of atherosclerosis.

Peptide analogs with high P‐selectin affinity can be utilized for targeting. Synthesized via anti‐Lewis antibodies and peptide phage libraries, IELLQAR is a peptide akin to selectin ligands. This mimetic effectively impedes the interaction between E‐, P‐, and L‐selectins and their sLeX ligands.^[^ ^69^ ^]^ Ye et al. reported that IELLQAR peptide analogs, which are selectin ligands, are highly inhibitory to P‐selectin binding to monocytes and can inhibit monocyte adhesion to the endothelium.^[^ ^75^ ^]^ Constantinescu et al. coupled a P‐selectin binding peptide (sequence H_2_N‐CKKKKLVSVLDLEPLDAAWL‐COOH) with a functionalized polyethylene glycosylated phospholipid to obtain targeting cationic liposomes that efficiently package and deliver siRNA to activated EC for use as specific delivery of siRNA thereby knocking down mRNA expression of target genes.^[^ ^70^ ^]^ In the same vein, Mocanu et al. used the same targeting strategy to develop lipid complexes (Psel‐lipo / shRAGE) carrying advanced glycosylation end products (RAGE) and short hairpin RNA (shRNA) to suppress the manifestation of RAGE.^[^ ^71^ ^]^ The targeted complexes demonstrated specific and efficient accumulation in the aorta of the ApoE^−/−^ mice. This led to the inhibition of atherosclerotic plaque development by decreasing RAGE protein expression in the aorta through the downregulation of NF‐кB and TNF‐α expression.

### 2.5 MCP‐1 specific targets

MCP‐1, alternatively known as CCL2, assumes a pivotal role in orchestrating the movement of inflammatory monocytes from the bone marrow and bloodstream to atherosclerotic plaques.^[^ ^76^ ^]^ This is achieved through its binding to its specific receptor CCR2. Monocyte migration is controlled by a concentration gradient of MCP‐1.^[^ ^77^ ^]^ Promisingly, strategies aimed at inhibiting the secretion of MCP‐1 and subsequently impeding monocyte chemotaxis hold the potential for effectively treating atherosclerosis by preventing the early development of plaques. Site‐specific delivery via ligand‐modified binding can provide a stable route to avoid damage to normal tissues and improve therapeutic efficiency due to internal body filters such as the liver, kidneys, and lymph nodes.^[^ ^77^ ^]^

Mog et al. identified a 4‐mer short peptide fragment (Cys‐Cys‐Thr‐Val, CCTV) presented on the surface of nanoparticles, consisting of two short peptides of cysteine, threonine, and valine, that exhibited potent MCP‐1 receptor‐targeting antagonist effects.^[^ ^78^ ^]^ CCTV exhibits a strong affinity towards CCR2 and binds to receptor proteins in a dosage‐dependent fashion through the *N*‐terminus of its cysteine residues, a structural feature absent in the free tetrapeptide form. Unlike cargo carrier‐directed drug delivery vehicles, nanoparticles themselves act as therapeutic agents, enhancing monocyte migration and resolving secondary inflammatory responses with significant downregulation of NF‐kB activity, but with no apparent effect on mediators of primary immune responses. Chin et al. used the targeting action of MCP‐1 peptides to dope Col‐1 peptides to develop peptide amphiphilic micelles (MCG PAM) with monocyte binding, collagenase inhibition, and gadolinium modification.^[^ ^79^ ^]^ Previous studies on atherosclerosis‐associated permeable arterial endothelium noted that leaky endothelial tight junctions span a range of approximately 20∼1330 nm.^[^ ^80^ ^]^ Considering the size of MCG PAMs is below 20 nm, these micelles can traverse malfunctioning endothelium, allowing for passive plaque targeting as well as active targeting via the CCR2 receptor. Through the MCP‐1 peptide‐binding motif, MCG PAM binds to monocytes and VSMCs in vitro, enabling targeting and imaging of atherosclerotic plaques and providing therapeutic collagenase inhibition, allowing for fibrous cap thickening and enhanced plaque stability.

## 3 TARGETING MOLECULES IN MACROPHAGE POLARIZATION AND FOAMY MACROPHAGES

After infiltrating the endothelium, monocytes undergo a process of differentiation, giving rise to two distinct types of macrophages: pro‐inflammatory types (M1 types^[^ ^81^ ^]^) and anti‐inflammatory types (M2 types^[^ ^82^ ^]^). This transformation is facilitated by various factors present in the local environment,^[^ ^83^ ^]^ such as Granulocyte‐macrophage colony‐stimulating factor (GM‐CSF), platelet factor 4, chemokines, and macrophage colony‐stimulating factor (M‐CSF).^[^ ^84^ ^]^ Once differentiated, these macrophages respond to specific stimuli including lipopolysaccharide (LPS), IFN‐c, and IL‐4, causing them to polarize accordingly.^[^ ^85^ ^]^ The activated macrophages proceed to internalize ox‐LDL in the endosomes by expressing specific scavenger receptors (SRs) including SR‐A, CD36, and CD47.^[^ ^86^ ^]^ When ox‐LDL is taken up by the body, it can cause the formation of foam cells that are rich in lipids. Macrophages engulf large amounts of oxidized lipids, which can transform them into foam cells, marking the early stages of atherosclerosis.^[^ ^2^ ^]^ The process of macrophage polarization and foamy macrophage formation involves a variety of up‐regulated molecules, such as CD44, and SR‐A, which can be used as targets for macrophage‐rich plaques.^[^ ^87^ ^]^ The overexpression of cell surface biomarkers on blood vessels and immune cells plays a crucial role in the advancement of atherosclerotic plaques, therefore, down‐regulating the expression process by targeting cell surface receptors is expected to alleviate the development of atherosclerosis.^[^ ^88^ ^]^

### 3.1 SR‐A specific targets

Current research on the regulation of scavenger receptors (SRs) in atherosclerosis has focused on five SR categories: SR‐A; SR‐B (CD25); SR‐E (LOX‐1); SR‐G (CXCL1); and SR‐J (RAGE).^[^ ^89^ ^]^ The activation of macrophages is a prominent early pathological alteration observed in atherosclerosis which overexpress SR‐A on their surface and are not expressed in normal vessel wall cells. Dextran sulphate (DS) is biocompatible, biodegradable, and safe, and it recognizes and binds to SR‐A.^[^ ^90^ ^]^ Therefore, suggested nanoplatforms exhibit selective targeting towards activated macrophages, leading to their preferential accumulation at the site. In addition, DS competitively inhibits the internalization of ox‐LDL by blocking SR‐A, thereby preventing its endocytosis.^[^ ^91^ ^]^

**FIGURE 2:**
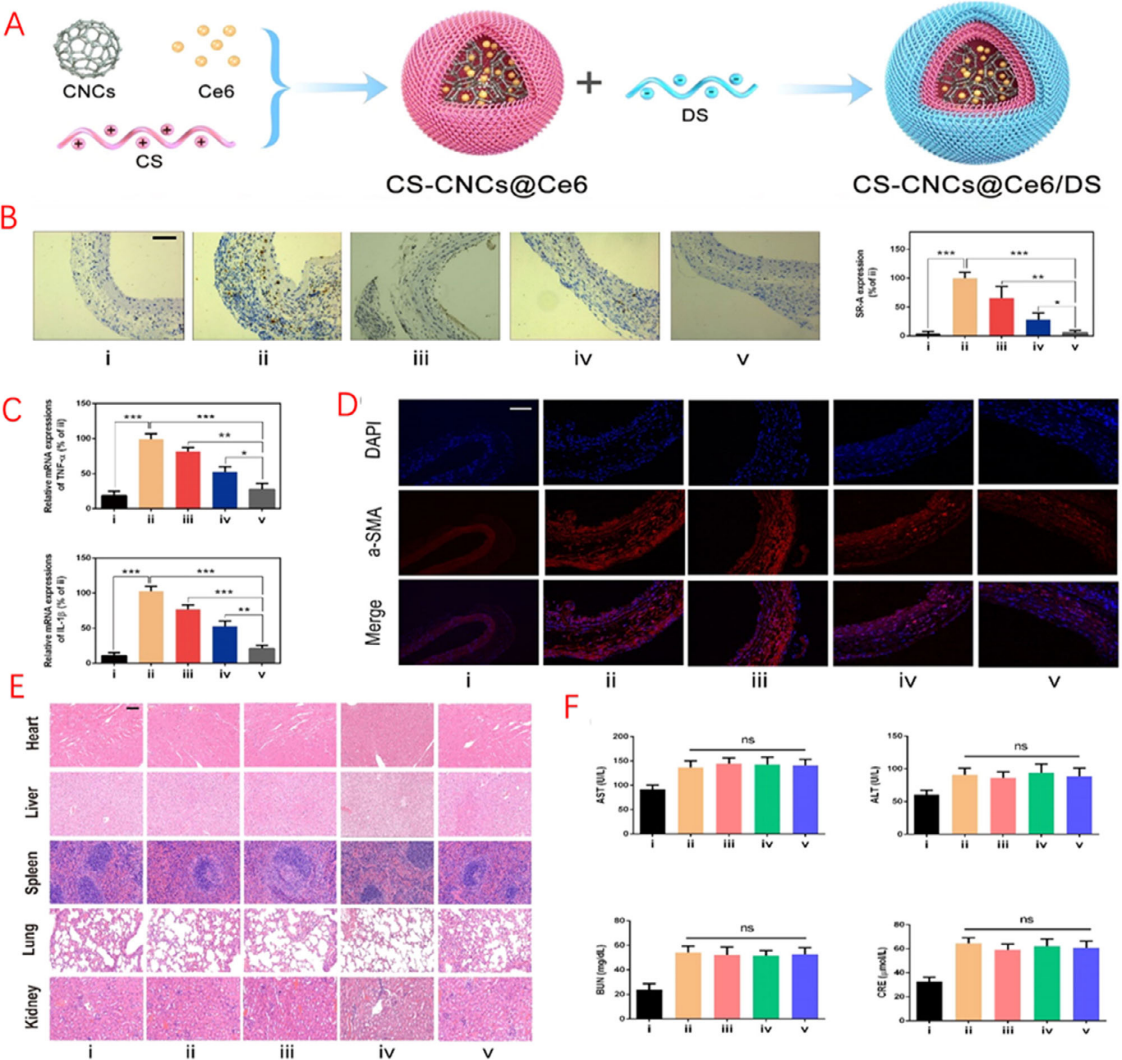
Nanocomplex CS‐CNCs@Ce6/DS with SR‐A targeting to alleviate atherosclerosis. (A) Schematic diagram of the preparation of NPs. (B–F) Mechanistic studies on ApoE^−/−^ mice. B) Immunohistochemical staining of carotid arteries. C) Expression of TNF‐α and IL‐1β mRNA in carotid arteries. D) Immunofluorescence staining of carotid arteries. E) H&E stained images of the heart, liver, spleen, lungs and kidneys. F) Levels of biochemical parameters of serum. Groups: (i) Control + saline + L808/633; (ii) AS + saline + L808/633; (iii) AS + CS‐CNCs@Ce6 + L808/633; (iv) AS + CS‐CNCs@Ce6/DS + L633/808; and (v) AS + CS‐CNCs@Ce6/DS + L808/633. Adapted with permission.^[^ ^93^ ^]^ Copyright 2021, American Chemical Society.

Ye et al. accomplished the successful synthesis of ultra‐low temperature enzyme inhibitors utilizing Fe‐PFH‐polylactide‐glycolic acid/chitosan‐DS.^[^ ^92^ ^]^ This innovative approach involved the combination of perfluoro hexane (PFH), a phase transition material, and DS, which specifically targets SR‐A. Leveraging the property of PFH to undergo phase transition upon exposure to low‐intensity focused ultrasound (LIFU) irradiation offers the potential to facilitate ultrasound imaging for the identification and control of unstable plaques in atherosclerosis. The findings indicate that in an isolated atherosclerotic plaque model of high cholesterol‐induced ApoE^−/−^ mice, NPs selectively accumulate in the aortic region activating macrophage SR‐A expression sites. Liu et al. used carbon nanocages, chitosan, and Chlorin e6 encapsulated to form a nanocarrier (Figure 2), and allowed the outermost layer to adsorb DS using electrostatic interactions to form the therapeutic nano‐complex CS‐CNCs@Ce6/DS.^[^ ^93^ ^]^ CNCs possess notable drug‐loading capacity because of their intrinsic hollow structure, but they exhibit limited hydrophilicity. Conversely, the application of chitosan (CS) coating notably augments their hydrophilicity and dispersibility in aqueous solutions, forming CS‐CNC. Through electrostatic interactions, DS binds to the surface of CS‐CNC by engaging its negatively charged sulphate group with the positively charged amine group of CS. The nano‐complex targeted towards SR‐A demonstrates specific recognition and attachment to activated inflammatory macrophages within atherosclerotic plaques, facilitating their uptake by these cells. This mechanism aids in decreasing the release of pro‐inflammatory cytokines and inhibiting the proliferation and migration of VSMCs. As a result, the nano‐complex contributes to the stabilization and diminishment of atherosclerotic plaques, thereby effectively impeding the advancement of atherosclerosis. This mechanism reduces the release of pro‐inflammatory cytokines, inhibits VSMC proliferation and migration, and contributes to plaque stabilization and diminishment, effectively impeding the progression of atherosclerosis.

### 3.2 CD9 specific targets

CD9, a cell surface glycoprotein, consists of four transmembrane structural domains and plays a pivotal role in regulating various cellular activities such as cell migration, proliferation, and adhesion.^[^ ^94^ ^]^ Elevated expression of CD9 has been observed in the human aorta and coronary arteries, particularly in regions affected by atherosclerotic lesions.^[^ ^95^ ^]^ By modifying the surface of CD9 antibodies, it becomes possible to achieve targeted delivery of loaded cargo specifically to atherosclerotic plaques that overexpress CD9. Additionally, this surface modification aids in inhibiting the progression of cellular senescence in atherosclerosis. Pham et al. developed a precise and specific nanosystem in which rosuvastatin (RSV) was loaded in CD9‐modified mesoporous silica nanoparticles (MSNs).^[^ ^96^ ^]^ MSNs have good biocompatibility, large pore size, and small dimensions, and therefore are commonly used to deliver therapeutic drugs (e.g. small molecule drugs, enzymes, genes (DNA or RNA), and diagnostic comparative reagents) to the permeability and retention of tumour regions and to minimize the systemic side effects of anticancer drugs.^[^ ^97^ ^]^ RSV is safeguarded within the porous domain of MSNs, subsequently enveloped using polyglutamic acid (PGA), poly‐l‐lysine (PLL), and hyaluronic acid (HA), culminating in the surface attachment of CD9 antibodies. Plaque hyaluronidase (HAase) weakens the intervening HA‐anchored shell, allowing the CD9 monoclonal antibody (mAb) to be released extracellularly once the HA coating is dissociated. The PGA and PLL inner layer outside the mesoporous core enhances stability, reduces uptake into the reticuloendothelial system, prevents rapid drug leakage, inhibits plasma protein‐repellent solubility, and prolongs MSN circulation time due to the ‘steric stabilization’ effect. The nanoparticles demonstrate a significant increase in cellular uptake, effectively reduce ROS levels, induce the production of TNF‐α and IL‐6, and mitigate the senescence process, consequently enhancing cell viability. These nanoparticles exhibit effective targeting capabilities towards regions affected by senile lesions, thereby mitigating the advancement of atherosclerosis in ApoE^−/−^ mice.

### 3.3 CD36 specific targets

The CD36 receptor, a vital transmembrane protein member of the class B scavenger receptor family, assumes a pivotal role in governing the entry of cholesterol into cells.^[^ ^98^ ^]^ CD36 plays a pivotal role in driving the transformation of macrophages into foam cells following the phagocytosis of ox‐LDL, positioning this receptor as a critical regulatory target in the management of atherosclerosis.^[^ ^99^ ^]^

Anti‐CD36 binds specifically to CD36 and can be used in the diagnosis^[^ ^98^, ^100^ ^]^ and treatment^[^ ^101^ ^]^ of AS. Wang et al. developed a novel approach by conjugating Anti‐CD36 to modified composite mesoporous silica nanoparticles (CMSN) to enhance the targeting capabilities for delivering the anti‐inflammatory drug SRT1720.^[^ ^101^ ^]^ In this study, the surface of CMSN was engineered with AntiCD36 to selectively target CD36, facilitating the monitoring and management of AS plaques. The efficacy of CMSN@SRT@Anti release was more pronounced in a neutral or slightly acidic milieu, possibly due to the favourable solubility of SRT1720 under mildly acidic conditions. Given the acidic microenvironment of AS lesions, nanomaterials demonstrating heightened drug release proficiency in acidic settings hold significant therapeutic promise. The findings revealed that CMSN@SRT@Anti, with its enhanced targeting ability, significantly improved the therapeutic efficacy of SRT1720 in treating atherosclerosis.

KOdiA‐PC, identified as 1‐(palmitoyl)−2‐(5‐keto‐6‐octenyl) phosphatidylcholine, a notable variant of oxidized phosphatidylcholine (PC) found within ox‐LDL, displays a robust attraction to the CD36 receptor, which plays a crucial role in the identification and internalization of ox‐LDL by endosomal macrophages.^[^ ^102^ ^]^ Zhang et al. achieved the successful synthesis of nanoparticles, referred to as Enano, which were efficiently loaded with epigallocatechin gallate (EGCG).^[^ ^103^ ^]^ A prior investigation exhibited that chitosan‐coated nanostructured lipid carriers (CSNLC) loaded with EGCG elevated the stability of EGCG. However, the presence of substantial chitosan and triglyceride content in these nanocarriers could potentially elevate blood glucose and triglyceride levels if administered to humans or research animals.^[^ ^104^ ^]^ Consequently, chitosan was eliminated, triglyceride was substituted with (+) alpha (α)‐tocopherol acetate and KOdiA‐PC was introduced as a CD36‐targeted ligand in the new formulation, resulting in ligand EGCG nanoparticles (L‐Enano) designed for precise conveyance of EGCG to intimal macrophages. Diverging from macrophages in other tissues and organs, intimal macrophages exhibit heightened expression of the CD36 scavenger receptor, thereby amplifying their capacity for the identification and binding of ox‐LDL.^[^ ^105^ ^]^ The administration of this formulation significantly decreased the synthesis of MCP‐1, TNF‐α, and IL‐6 in mouse macrophages. Additionally, it resulted in a decrease in the surface area of aortic arch lesions in LDLr^−/−^ mice. Since L‐Enano targets endothelial macrophages, it is less absorbed by the liver compared to natural EGCG and Enano. Dhanasekara et al. used soy phosphatidylcholine to synthesize liposome‐like nanoparticles and subsequently incorporated KOdiA‐PC onto their surface to produce ligand‐functionalized nanoparticles (L‐NPs)^[^ ^106^ ^]^. L‐NPs mimic the lipid whisker pattern akin to oxPCs, wherein the hydrophilic head and truncated sn‐2 oxidative moiety of oxPCs project from the surface of the phospholipid membranes of ox‐LDL. In contrast, L‐NPs interact with macrophage CD36 receptors through the externally protruding KOdiA‐PC. The robustness of this investigation stems from the integration of KOdiA‐PC as a CD36‐targeted ligand, facilitating the creation of biocompatible and biodegradable L‐NPs devoid of any chemical conjugation or modification procedures. The precision of targeting has been validated through experimentation with mouse and human macrophages, along with animal models of atherosclerosis. The data showed that L‐NPs accumulated 1.4 times more than NPs in the aortic lesion region. Craparo et al. incorporated KOdiA‐PC into the initial organic phase, resulting in the formulation of a powdered compound comprising polymeric nanoparticles embedded with mannitol (Man) and encapsulating Rap.^[^ ^107^ ^]^ This innovative approach aims to selectively target atherogenic macrophages for parenteral administration, aiming to address atherosclerosis. The drug was encapsulated in particles measuring less than 100 nm, effectively in an amorphous state. Furthermore, the lyophilization process, conducted in the presence of Man, facilitated the reconstitution of the formulation, yielding properties nearly identical to the original, rendering it appropriate for parenteral administration. The powdered formulation exhibited robust stability during storage and, upon reconstitution in a physiological medium, demonstrated the capability to shield the encapsulated drug from degradation while ensuring controlled release. Even at elevated concentrations, the nanoparticles exhibited no cellular toxicity, as affirmed by uptake assays that verified the internalization of the nanoparticles.

### 3.4 CD44 specific targets

CD44 is a receptor located on the cell surface, which exhibits higher expression levels on cells within atherosclerotic plaques. Its primary natural binding partner is a polysaccharide known as HA.^[^ ^108^ ^]^ In the context of inflammation and the presence of inflammatory cues such as TNF‐α, CD44 undergoes sulfation, inducing structural alterations within the protein and prompting its conversion into a high‐affinity configuration that effectively binds to HA.^[^ ^109^ ^]^ The targeting ligand HA binds to CD44, an upregulated cell adhesion molecule in damaged endothelium in atherosclerotic lesions, and can be used for plaque targeting.^[^ ^110^ ^]^ In addition, HAase production is highly abundant in the region of atherosclerotic plaques, leading to the subsequent exposure of encapsulated nanoparticles.^[^ ^111^ ^]^ Therefore, many researchers have used HA modification to achieve drug delivery in atherosclerotic plaques by targeting intraplaque macrophages, such as statins (atorvastatin,^[^ ^112^, ^113^ ^]^ simvastatin,^[^ ^111^, ^114^, ^115^ ^]^ rosuvastatin^[^ ^116^ ^]^), and anticoagulants (heparin^[^ ^117^ ^]^).

**FIGURE 3:**
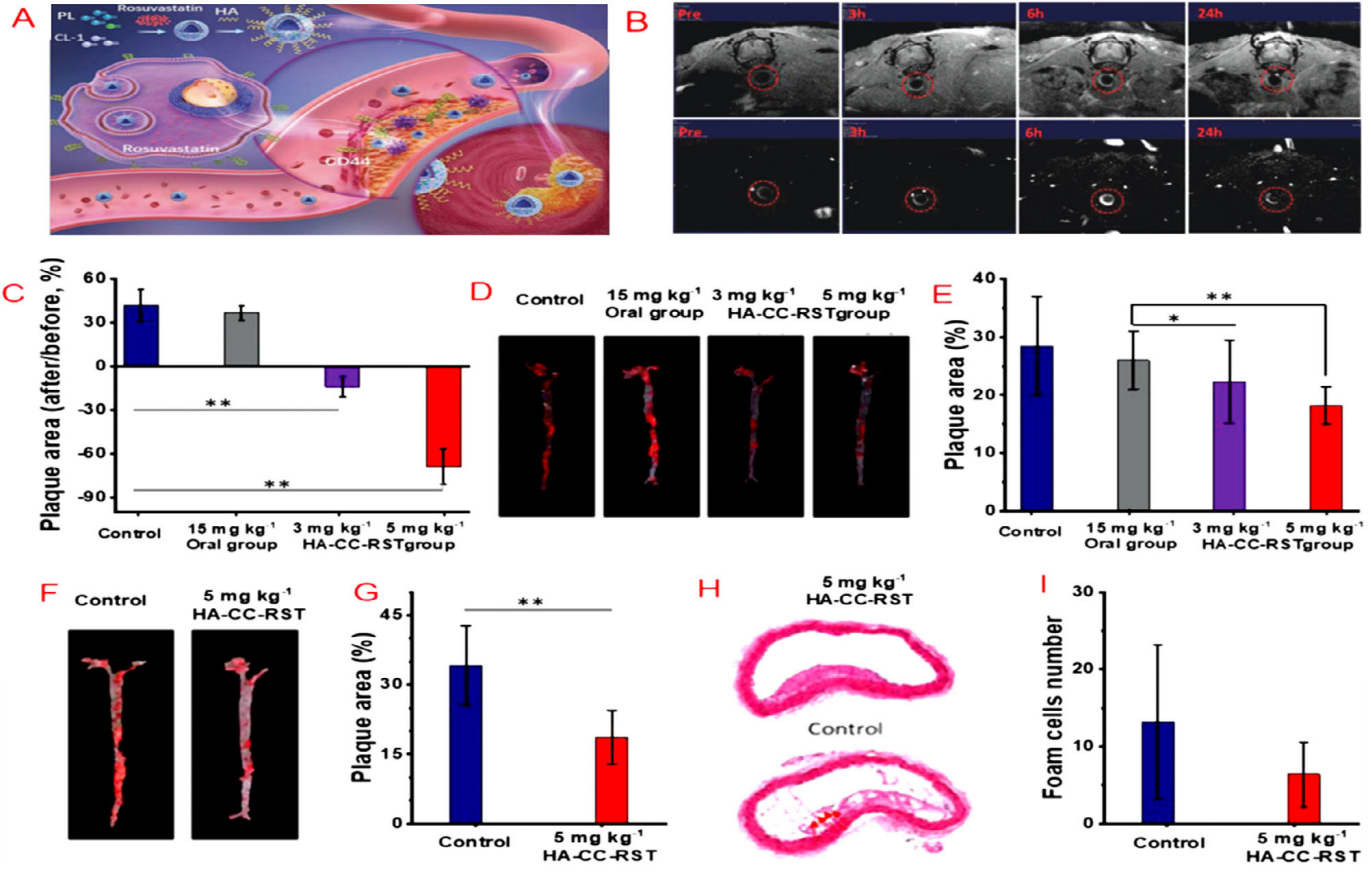
Hyaluronic acid‐targeted nano‐agent HA‐CC‐RST for simultaneous imaging and treatment of advanced atherosclerosis. (A) Schematic representation of nanoparticle targeting. (B) Imaging of hyaluronic acid‐modified neuronal gadodiamide (HA‐CC‐Gd) in a moderate aortic atherosclerosis model. (C–I) In vivo efficacy of HA‐CC‐RST in reversing plaque models of different severity. C,D) Plaque area measurement and oil‐red O staining in a carotid atherosclerosis model. E,F) Quantification of plaque area and oil‐red O staining in a moderate aortic atherosclerosis model. G–I) Quantification of plaque area and H&E staining of aortic cross‐sections in an advanced aortic atherosclerosis model with histological analysis to determine the number of foam cells. Adapted under the terms of the CC BY 4.0 DEED license.^[^ ^116^ ^]^ Copyright 2022, The Authors.

Hossaini Nasr et al. designed a new HA‐atorvastatin (ATV) coupling (HA‐ATV‐NPs).^[^ ^112^ ^]^ To enhance the effectiveness of drug delivery, this novel approach utilizes the hydrophobic properties of a drug called ATV. By bonding ATV with HA, a new compound is formed that can self‐assemble into nanoparticles in water, with ATV serving as the hydrophobic core. When exposed to macrophages with CD44 receptors, the HA‐ATV nanoparticles mimic the natural process of inflammation by attracting these cells to plaque sites. This enables the nanoparticles to selectively accumulate within plaques and deliver ATVs to these targeted locations. In vitro tests showed that the nanoparticles were more effective in reducing inflammation in macrophages than unencapsulated ATVs. In vivo tests conducted on ApoE^−/−^ mice also demonstrated a significant reduction in inflammation within atherosclerotic plaques. Additionally, this approach holds the potential to serve as a versatile platform technology, suitable for the targeted conveyance of alternative statins or anti‐inflammatory agents geared towards attenuating inflammation linked to plaques. Given that several of these compounds can serve as the hydrophobic core within HA nanoparticles, the versatility of this strategy remains promising. Similarly, Obaid et al. prepared a nano platform called SIM/ZIF‐8@HA by encapsulating simvastatin (SIM) within zeolitic imidazolate framework‐8 (ZIF‐8)^[^ ^118^ ^]^ and subsequently coating it with a HA layer.^[^ ^111^ ^]^ The NPs demonstrated the ability to effectively inhibit the proliferation of VSMCs while exhibiting excellent biocompatibility. Ma et al. developed HA‐modified mixed liposomal nerves (CCs) encapsulated with rosuvastatin (HA‐CC‐RST) to reverse the efficacy of an in vivo model of ApoE^−/−^ rats with varying severity of plaque.^[^ ^116^ ^]^ CCs are effective drug delivery systems due to their stable silica‐like surfaces, biocompatibility, and ability to encapsulate both hydrophobic and hydrophilic drugs. In this study, RST was loaded into CC membranes using a thin‐film hydration method, while hydrophilic Gd was encapsulated in the inner water phase. The incorporation of an inorganic polysiloxane network on the CC surface effectively protected the internal lipid bilayer structure and reduced drug leakage. The administration of HA‐CC‐RST at doses 50‐fold lower than oral RST demonstrated remarkable efficacy in reversing atherosclerosis (Figure 3). This treatment led to a substantial reduction in both plaque area and volume, with a reduction of 56.3% and 78.7%, respectively. These results indicate a significant enhancement in drug delivery efficiency. Liu et al. successfully prepared multifunctional nanoparticles named H‐CuS@DMSN‐NC‐HA,^[^ ^117^ ^]^ which utilized dendritic mesoporous silica encapsulated with water‐soluble copper sulphide nanoparticles. The purpose of designing this nano platform was to utilize electrostatic adsorption for the incorporation of the anticoagulant drug heparin. Moreover, the nano platform underwent modification with oxidized HA, serving to hinder untimely drug release and enhance targeted delivery to inflammatory macrophages located at the lesion site. Research has demonstrated the nanoparticles' favourable biocompatibility and photothermal properties for the targeted elimination of macrophages and thrombi. Also, it exhibited superior chemotherapeutic‐photothermal synergistic anti‐atherosclerotic therapeutic efficiency compared to treatment alone.

Sonodynamic therapy (SDT)^[^ ^119^ ^]^ and photothermal therapy (PTT)^[^ ^120^ ^]^ are two effective methods for the treatment of atherosclerotic plaques. Cao et al. successfully developed copper sulphide/titanium oxide heterostructured nanosheets (CuS/TiO2) modified with HA and polyethylene glycol, referred to as HA‐HNSs.^[^ ^121^ ^]^ These nanosheets served as effective agents for acoustic kinetic and photothermal therapies in the synergistic treatment of early‐stage atherosclerotic plaques. The combined approach of SDT and PTT using HA‐HNSs induced apoptosis of pro‐inflammatory macrophages, resulting in a consistent reduction in regions positive for the pro‐inflammatory cytokines TNF‐α and IL‐6. By combining this synergistic therapeutic approach, the advancement of early atherosclerotic plaques was effectively halted through the elimination of diseased macrophages and the reduction of inflammation.

### 3.5 Mannose receptor‐specific targets

The expression of the mannose receptor (MR) is limited in resting macrophages, but it becomes significantly upregulated in M2 macrophages, presenting an attractive prospect for targeting purposes.^[^ ^122^ ^]^ MR possesses a C‐type lectin‐like structural domain (CTLD) that enables it to recognize and internalize mannose, fucose, and *N*‐acetylglucosamine located at the terminus of polycarbonates.^[^ ^123^ ^]^ This integral membrane protein serves as a molecular gateway for these specific carbohydrate molecules. Chen et al. investigated a newly developed ligand called MR‐targeted ligand (MRTL), which demonstrated exceptional selectivity for the MR and explored the feasibility of selective targeted nanoparticle delivery.^[^ ^124^ ^]^ The MRTL consisted of two mannose molecules connected by a precisely engineered polyethylene glycol linker comprising 12 units of uniform size and distribution.^[^ ^125^ ^]^ The findings indicate that nanoscale macromolecular carriers equipped with polymeric mannose‐targeting ligands hold promise for targeted delivery to activated M2 macrophages. This approach enables specific and selective targeting of these macrophage subtypes.

## 4 TARGETING VASCULAR SMOOTH MUSCLE CELLS AND VULNERABLE ATHEROSCLEROTIC PLAQUES‐RELATED MOLECULES

VSMCs perform a pivotal role in the progression of atherosclerotic lesions into complex plaques.^[^ ^126^ ^]^ On the one hand, they are guided by growth factors released by the activated platelets^[^ ^127^ ^]^ to migrate from the mid‐membrane to the intima.^[^ ^128^ ^]^ In response to cytokines such as IL‐1β and lipid buildup, VSMCs undergo a phenotypic shift from a contractile state to a synthetic state.^[^ ^137^ ^]^ This shift activates NF‐κB, leading to the release of inflammatory molecules like IL‐6.^[^ ^129^ ^]^ VSMCs internalize ox‐LDL to form foam cells by expressing a variety of receptors associated with cholesterol uptakes, including SR‐A, and LOX‐1.^[^ ^130^ ^]^ It also produces M‐CSF, promoting the expansion of macrophage populations within the atherosclerotic plaque.^[^ ^131^ ^]^ Ultimately, promoting the production of large numbers of foam cells. Even though foam cells can excrete cholesterol via the transport proteins ABCA1 and ABCG1,^[^ ^132^ ^]^ they frequently undergo apoptosis or necrosis, increasing the likelihood of lesion rupture.^[^ ^133^ ^]^ On the other hand, VSMCs accumulate around a lipid pool composed of a ‘necrotic nucleus’, synthesizing and secreting collagen to form an extracellular matrix, thus acting as a structural barrier in the formation of the fibrous cap, conferring stability to the atherosclerotic plaque and reducing the risk of rupture, with a typical atherosclerotic plaque.^[^ ^134^ ^]^ Expanding VSMCs produce extracellular matrix (ECM) elements like fibrin and collagen, contributing to the formation of the protective fibrous cap encasing atherosclerotic plaques. In addition, inflammatory mediators such as IL‐1β, and TNF‐α exert a detrimental effect on collagen production and stimulate the expression of matrix metalloproteinases (MMPs).^[^ ^135^ ^]^ The increased activity of MMPs promotes the degradation of ECM components, particularly collagen, thereby weakening the fibrous cap.^[^ ^136^ ^]^ As a consequence, the plaque becomes vulnerable to rupture, posing a significant risk for adverse cardiovascular events.^[^ ^137^ ^]^ The interplay among the constituents of exposed atherosclerotic plaque, platelet receptors, and coagulation factors ultimately triggers platelet activation, and aggregation, culminating in the subsequent generation of overlaid thrombi.^[^ ^138^ ^]^ Currently, nanoparticles targeting Profilin‐1, Osteoblastin, Integrin αvβ3, or Type IV collagen have been designed to specifically target VSMCs or vulnerable atherosclerotic plaques (VASP) to improve the accuracy of diagnosis and treatment.

### 4.1 Profilin‐1 specific targets

Abundant in atherosclerotic VSMCs, profilin‐1 participates in the reconfiguration of cytoskeletal structures.^[^ ^139^ ^]^ Investigations have indicated that heightened profilin‐1 expression contributes to the onset of atherosclerosis by influencing the migration and proliferation of VSMCs.^[^ ^140^ ^]^ Hence, profilin‐1 has been identified as a molecular indicator of atherosclerosis,^[^ ^141^ ^]^ presenting itself as a promising target for the diagnosis and therapeutic intervention of this condition. Zhang et al. developed a novel approach by combining cyclodextrin nanoparticles (NPs) with profilin‐1 antibody (PFN1) to specifically target VSMCs within atherosclerotic plaques. These NPs were further integrated with the anti‐inflammatory drug Rap, resulting in the formulation known as RAP@PFN1‐CD‐MNPs.^[^ ^142^ ^]^ For enhanced colloidal stability under physiological conditions, it is crucial to extend circulation time in the bloodstream while minimizing in vivo toxicity. Magnetite nanoparticles are often surface‐functionalized with biocompatible polymers like dextran or polyvinyl alcohol.^[^ ^143^ ^]^ In this research, the authors employed polyethylene glycol(PEG)‐PEI to modify superparamagnetic iron oxide (SPIO) NPs due to its favourable solubility in aqueous solutions and its capability to extend circulation duration within the bloodstream.^[^ ^144^ ^]^ The study suggests that these nanoparticles have the potential as a contrast agent in visualizing atherosclerotic lesions and delivering therapeutic agents effectively in low pH environments for atherosclerosis treatment.

### 4.2 OPN specific targets

Osteoblastin (OPN) is a secreted phosphorylated glycoprotein that is recognized as a biomarker for the phenotypic transition in VSMCs.^[^ ^145^ ^]^ Significantly higher OPN expression as well as enhanced macrophage recruitment was found in foam cells.^[^ ^146^ ^]^ Studies have indicated that OPN serves as a promising target for the detection of VASP.^[^ ^147^ ^]^ To enhance the sensitivity and specificity of nanoparticles, the surface can be modified by targeting ligands such as antibodies and peptides, thereby allowing for their precise recognition and binding.

**FIGURE 4:**
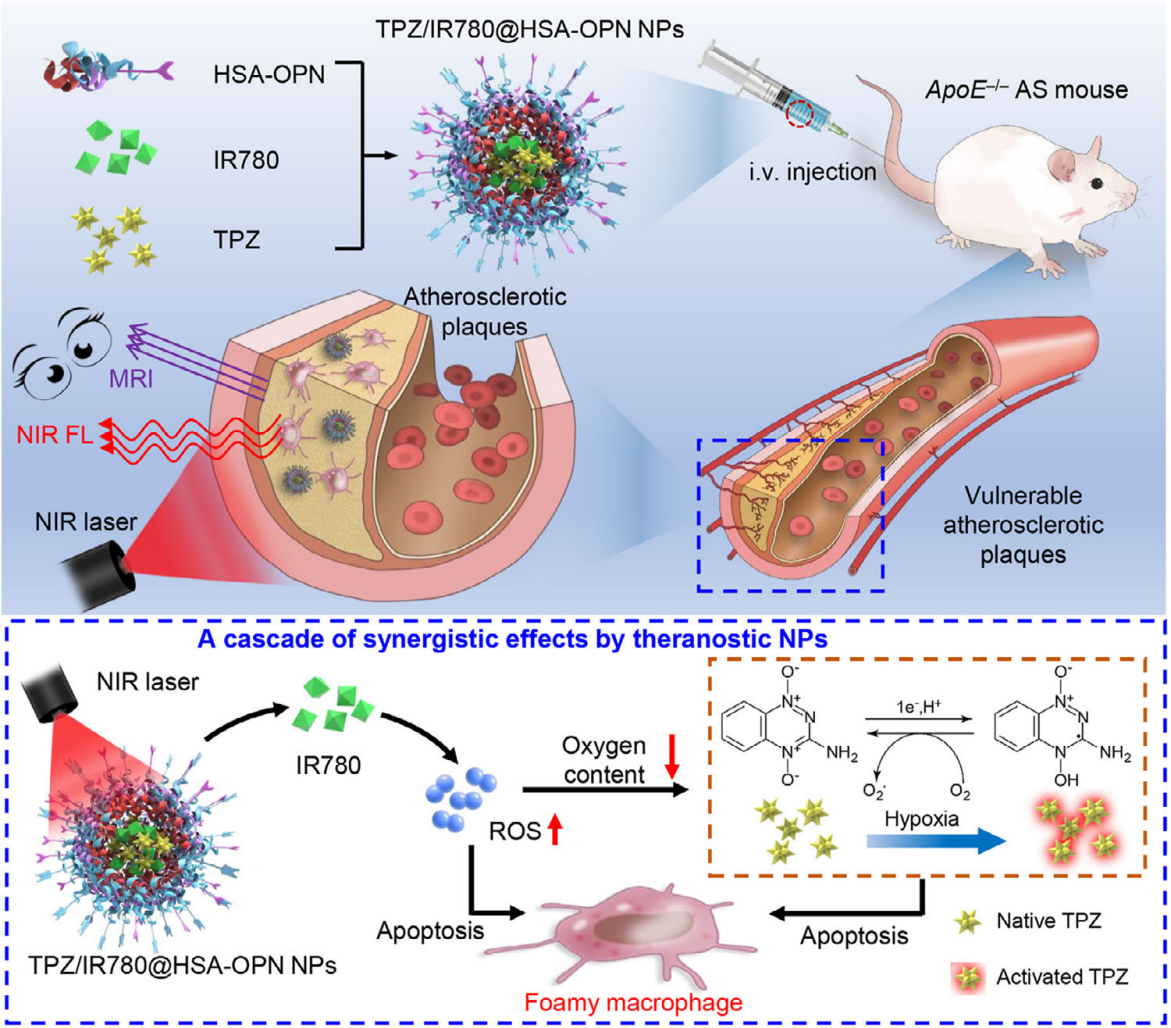
Theranostic nanoprobes (TPZ/IR780@HSA‐OPN) were developed to specifically target and regress VASPs. These nanoprobes act in response to local laser irradiation and are guided by fluorescence/MR imaging. They are composed of human serum albumin (HSA) attached to a peptide that targets osteopontin (OPN) and contains both IR780 photosensitizer and tirapazamine (TPZ), which is activated under hypoxic conditions. When these OPN‐targeted nanoparticles were introduced into mice with atherosclerosis, they showed high accumulation in VASPs, primarily because of the increased presence of OPN in certain carotid artery macrophages. Utilizing fluorescence and MR imaging for guidance, specific near‐infrared (NIR) laser exposure produced significant reactive oxygen species (ROS). Adapted under the terms of the CC BY‐NC‐ND license. ^[^ ^150^ ^]^ Copyright 2022, Chinese Pharmaceutical Association and Institute of Materia Medica, Chinese Academy of Medical Sciences.

Huang et al. designed and constructed nanoparticles (GW1516@NP‐OPN) coupled with anti‐OPN antibodies, which encapsulated agonists of PPARδ receptors GW1516, and demonstrated efficiently suppressing the migration and apoptosis of VSMCs induced by ox‐LDL.^[^ ^148^ ^]^ Suggestively, the experimental results show that antibody modification affects drug release from the nanoparticle system. Conjugation of anti‐OPN antibody leads to higher micelle density and covalent binding of micelles to antibody may alter the hydrophilic end structure and increase the size of the interstitial space leading to slower drug release from OPN‐targeted NPs than non‐targeted NPs. Ma et al. fabricated NPs (termed ICG/SRT@HSA‐pept NMs) by modifying human serum albumin (HSA) with a high‐affinity peptide derived from OPN and then incorporating the near‐infrared (NIR) fluorescent dye indocyanine green (ICG) along with the sirtuin 1 activator SRT1720.^[^ ^149^ ^]^ The study revealed that by targeting NMs, they specifically accumulate in the VASP region, allowing for precise detection of VASP. Moreover, in the presence of SRT1720, the NMs could activate intracellular Sirt1, which led to the induction of anti‐atherosclerotic effects by suppressing VSMCs phenotypic switching. This led to a significant improvement in the physiological size and plaque composition of VASP after two weeks of treatment. Similarly, Xu et al. synthesized TPZ/IR780@HSA‐OPN (Figure 4) nanoparticles through the conjugation of HSA with OPN‐targeting peptides, incorporating the photosensitizer IR780 and the hypoxia‐activated verapamil (TPZ).^[^ ^150^ ^]^ Animal studies demonstrated a significant reduction in plaque area and carotid stenosis after 2 weeks of treatment with the NPs.

### 4.3 Integrin αvβ3 specific targets

Integrin αvβ3 has an important role in platelet hemostasis and thrombosis.^[^ ^151^ ^]^ The upregulation of integrin αvβ3 in neovascularization within plaques is closely linked to the modulation of cell proliferation, migration, and cytokine activation, suggesting its potential as a target for identifying and treating susceptible plaques.^[^ ^151^ ^]^ The peptide cRGD possesses a specific binding affinity towards integrin αvβ3, allowing for targeted recognition and interaction, and targeting atherosclerotic plaques in ApoE^−/−^ mice.^[^ ^152^ ^]^

Kim et al. bound cRGD peptide to iron oxide nanoparticles (IONP) targeting the integrin αvβ3 to transport the anti‐inflammatory component (IL‐10) to plaques.^[^ ^153^ ^]^ Similarly, Li et al. targeted IL‐10 to atherosclerotic plaques via cRGD‐coupled liposomes (IL10‐cRGD‐ lip).^[^ ^154^ ^]^ They created DSPE‐PEG‐cRGD utilizing a thiol‐maleimide bond, enhancing liposomal targeting efficiency. The liposomes took the shape of large unilamellar vesicles (LUVs) which had a size ranging from 100 to 200 nm. LUVs are more stable during storage than small unilamellar vesicles (SUVs) as the latter may merge at temperatures below the lipid's phase transition point. The outcomes demonstrated that the targeted delivery facilitated by the nanocarriers led to the efficient localization of IL‐10 at the sites of plaque, effectively countering the generation of pro‐inflammatory cytokines within the lesion.^[^ ^153^ ^]^

**FIGURE 5:**
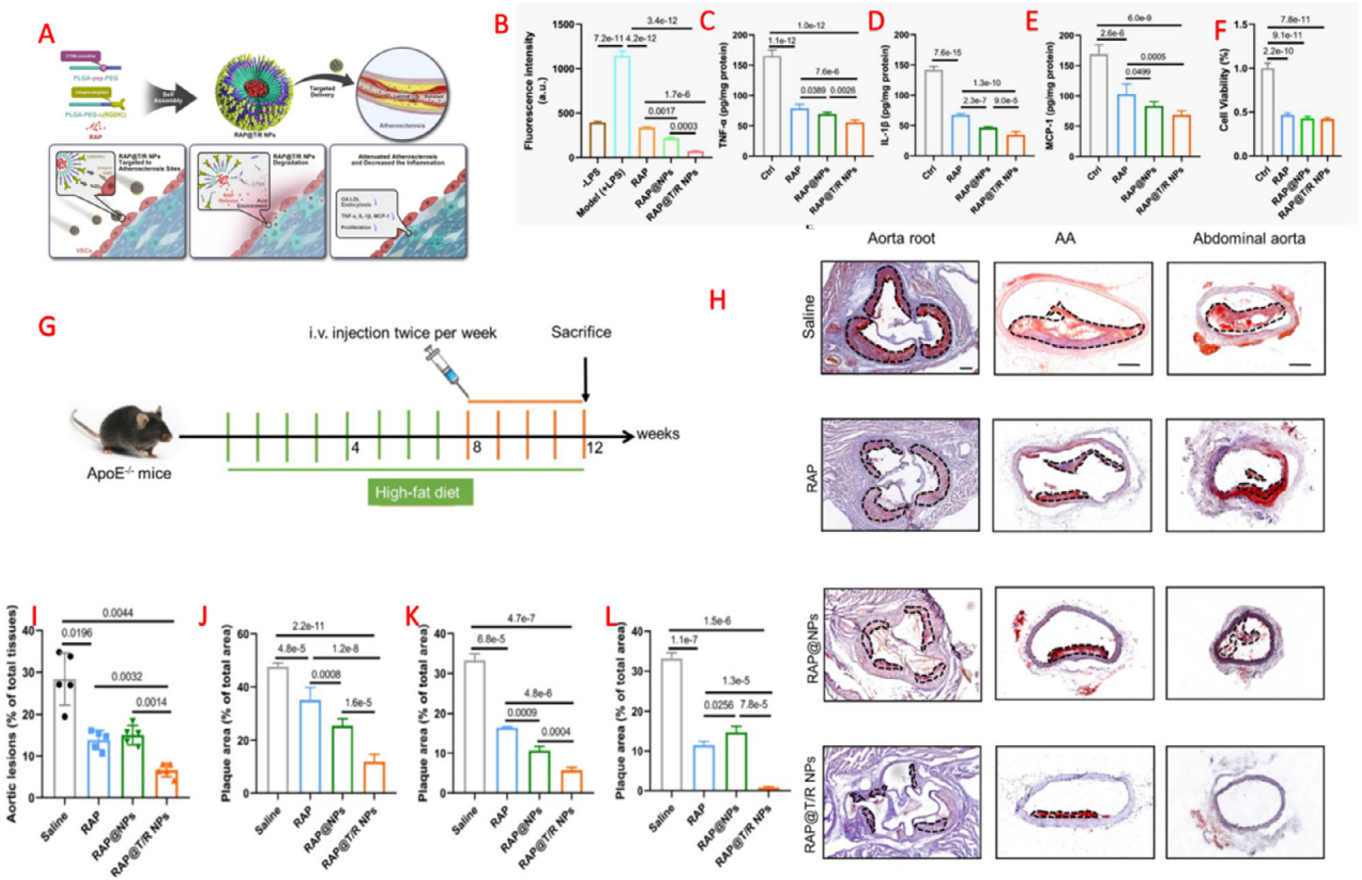
Integrin αvβ3‐targeted and CTSK‐responsive nanoparticles RAP@T/R NPs targeting atherosclerosis. (A) Schematic representation of NPs composition. (B–F) Evaluation of the in vitro anti‐inflammatory effect of NPs, (B) fluorescence intensity of DIR to evaluate cellular uptake, ELISA detection of inflammatory cytokines (C) TNF‐α, (D) IL‐1β, and (E) MCP‐1 in LPS‐treated RAW264.7 secretion, and (F) cell viability. (G) Schematic of in vivo experimental protocol. (H) Oil red staining of the aortic root, aortic arch (AA), and abdominal aorta. (I) Quantification of lesion area in aortic tissue. (J–L) Quantification of relative plaque area in sections of the (J) aortic root, (K) AA, and (L) abdominal aorta. Adapted under the terms of the CC BY 4.0 DEED license.^[^ ^155^ ^]^ Copyright 2022, The Authors.

Fang et al. constructed nanoparticles modified with c(RGDfC) were developed to selectively target integrin αvβ3 and tissue proteinase k (CTSK)‐sensitive nanoparticles (RAP@T/R NPs) (Figure 5) to regulate the localized release of rapamycin.^[^ ^155^ ^]^ The c(RGDfC) peptide's thiol group reacted with PLGA‐PEG‐MAL's maleimide group, forming PLGA‐PEG‐c(RGDfC). Modified hydrophilic PEG on nanoparticle surfaces created a hydration layer that prevented protein adsorption via steric repulsion, enhancing targeted drug delivery by evading recognition and clearance.^[^ ^156^ ^]^ PEGylated T/R NPs exhibited an extended 48‐h circulation time. To prevent liver toxicity from Rap accumulation,^[^ ^157^ ^]^ a stimuli‐responsive strategy was used, where CTSK‐responsive nanoparticles loaded with Rap reduced nonspecific release.^[^ ^158^ ^]^ T/R NPs rapidly released Rap in high concentrations of CTSK and acidic atherosclerotic microenvironments but had limited release elsewhere. These particles effectively suppressed the phagocytosis of ox‐LDL and cytokine secretion by macrophages, accumulating in both early and advanced atherosclerotic lesions.

### 4.4 Type IV collagen‐specific targets

Constituting approximately 50% of the basement membrane matrix, type IV collagen (Col‐IV) is a non‐fibrillar protein.^[^ ^159^ ^]^ Throughout the advancement of atherosclerosis, there is a notable augmentation in Col‐IV within atherosclerotic plaques.^[^ ^160^ ^]^ Recent research has highlighted the efficacy of Col‐IV‐targeting peptides as valuable ligands for nanoparticles aimed at targeting atherosclerosis.

In recent research, a collagen‐binding peptide was utilized to enhance the homing migration of Col‐IV‐targeted NPs toward atherosclerotic lesions. The Ac2‐26 peptide, through the *N*‐formyl peptide receptor 2, facilitated inflammation resolution, emulating an anti‐inflammatory response.^[^ ^161^ ^]^ Upon receptor activation in myeloid cells, the Ac2‐26 peptide mitigated lesion‐associated superoxide production, diminishing plaque necrosis and thereby stabilizing advanced atherosclerotic lesions with chronic and unresolved inflammation.^[^ ^162^ ^]^ Yu et al. harnessed the Peptide Ac2‐26 as a Col‐IV‐targeting ligand to functionalize NPs, optimizing formulation parameters including the length of PEG‐coated molecules.^[^ ^163^ ^]^ Experimental outcomes indicated that Col IV‐targeted nanoparticles (Col‐IV‐GW‐NPs) encapsulating the Liver X receptors(LXR) agonist GW3965 (GW) effectively reached atherosclerotic lesions. During the treatment, mice that were given Col IV‐GW‐NPs did not develop increased hepatic lipid biosynthesis or hyperlipidemia, unlike those that were given free GW. This indicates that Col IV‐GW‐NPs provide an improved delivery mechanism for LXR agonists to atherosclerotic lesions without any negative effects on hepatic lipid metabolism.

## 5 MULTI‐TARGETED COMBINATION THERAPY FOR ATHEROSCLEROSIS

Multi‐targeting strategies have been used to improve precise targeting and therapeutic efficacy at the site of atherosclerosis, and more and more researchers are working to construct nanomaterials that target two or three receptors or cytokines at the same time. Examples include CD44 with VCAM‐1^[^ ^164^, ^165^ ^]^ or E‐selectin,^[^ ^166^ ^]^ SR‐B1 with CD36^[^ ^167^ ^]^ or integrin αIIbβ3,^[^ ^168^ ^]^ CD47 and integrin α4/β^[^ ^169^ ^]^ dual‐targeted synergy therapy. Furthermore, Yan et al. formulated microbubbles (MBs) with triple targeting capabilities towards VCAM‐1, ICAM‐1, and P‐selectin, which can be used to monitor the onset and progression of AS or to assess the efficacy of drug interventions such as atorvastatin.^[^ ^170^ ^]^

**FIGURE 6:**
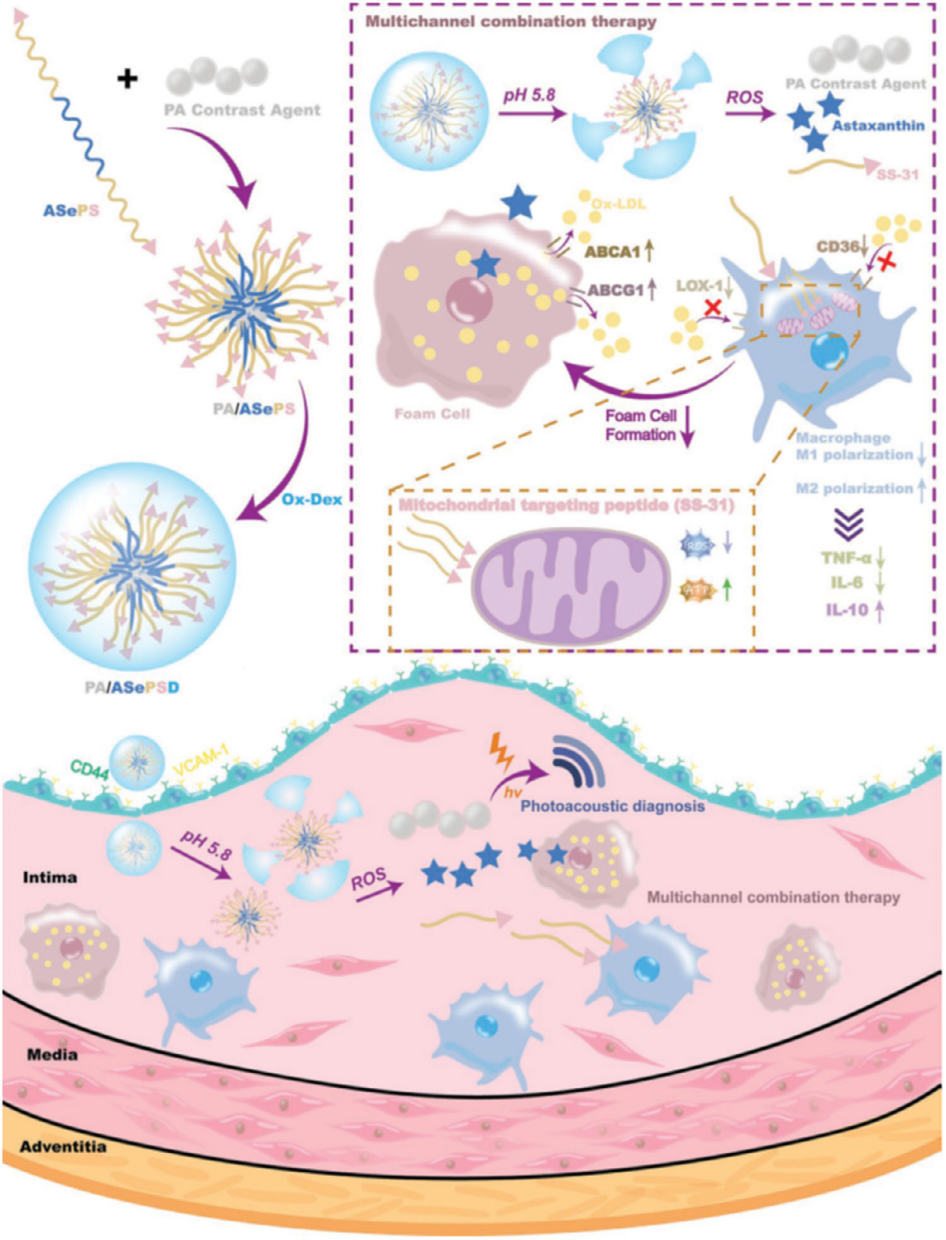
Nanoplatform PA/ASePSD targeting VCAM‐1 and CD44 for atherosclerosis diagnosis and treatment. The nanoparticles possess the ability for cascade targeting towards atherosclerotic plaques and cellular mitochondria, enabling a hierarchical response to the acidic microenvironment and elevated ROS levels. This response leads to the disintegration of the nanoparticles, subsequently triggering anti‐inflammatory effects and modulating the dynamic balance of cellular lipid flow. Additionally, the nanoparticles release a photoacoustic contrast agent, enabling targeted photoacoustic diagnosis of atherosclerosis and facilitating multi‐channel combination therapy. Adapted with permission.^[^ ^165^ ^]^ Copyright 2021, Wiley‐VCH.

Dual targeting systems have emerged as more potent alternatives to single receptor targeting systems, demonstrating a promising strategy to enhance the efficacy of atherosclerotic plaque targeting while minimizing unintended uptake by the liver.^[^ ^167^ ^]^ Xu et al. demonstrated the efficacy of dextran‐based nanoparticles for precise targeting of atherosclerotic lesions. These nanoparticles are selectively bound to VCAM‐1 and CD44 receptors found on compromised endothelial cells and macrophages, facilitating active targeting.^[^ ^164^ ^]^ Simultaneously, the nanoparticles successfully delivered the anti‐inflammatory drug prednisolone. Dextran, a naturally occurring branched glucose polymer known for its strong biocompatibility, exhibits a notable binding affinity with VACM‐1 and CD44 receptors that are specifically present in impaired endothelial cells. This characteristic empowers the nanoparticles with an active targeting ability toward atherosclerotic sites. Notably, within the atherosclerotic microenvironment characterized by elevated ROS levels and abundant lipids, poly(2‐(methylthio)ethyl methacrylate) (PMEMA) can transition from hydrophobic to hydrophilic states. This transformation prompts the nanoparticle structure to swell, ultimately facilitating the release of Prednisolone while concurrently aiding lipid removal, owing to enhanced interactions between lipids and cyclodextrins. On this basis, they further optimized the ability of dextran to target VCAM‐1 and CD44 and used π‐conjugated polymer (PMeTPP‐MBT) loaded with astaxanthin and Szeto‐Schiller 31 (SS‐31) peptide to form nanoparticles (PA/ ASePSD) (Figure 6).^[^ ^165^ ^]^ The nanoparticles exhibit the ability to achieve controlled release within the acidic plaque microenvironment, particularly under conditions of elevated levels of ROS. The SS‐31 peptide specifically targets macrophage mitochondria, leading to the inhibition of ROS production, restoration of mitochondrial function, and reduction of cholesterol inward flow. Simultaneously, astaxanthin amplifies the ABCA1/G1 expression within foam cells, working in synergy with the SS‐31 peptide to produce a collective anti‐inflammatory impact. Furthermore, employing the loaded photoacoustic contrast agent PMeTPP‐MBT allows for non‐invasive, real‐time diagnosis of early‐stage atherosclerosis. The results of the study indicate that treatment with PA/ASePSD nanoparticles in ApoE^−/−^ mice leads to a substantial upregulation of ABCA1/G1 protein expression within aortic plaques, while concurrently suppressing the expression of LOX‐1/CD36 at the plaque site. Chen et al. synthesized two micelle carriers, SCCF and SCTM, loaded with rapamycin using the specific binding of *N*‐acetylneuraminic acid (SA) to the E‐selectin receptor and the superior target binding ability of chondroitin sulphate to the CD44 receptor.^[^ ^166^ ^]^ ORO results confirmed the significant anti‐atherosclerotic therapeutic effect of both. Gao et al. introduced a novel approach called “Multifunctional Pathology Mapping Therapy Diagnostic Nanoplatform (MPmTN)” aimed at classifying plaques based on their pathology. This strategy involves the utilization of the PP1 peptide, which attaches to macrophage SR‐A receptors, along with the cRGD peptide that binds to activated platelet glycoprotein (GP) IIb/IIIa receptors.^[^ ^168^ ^]^ The MPmTNs are composed of poly(lactic acid‐glycolic acid) nanoparticles containing Fe_3_O_4_ as a contrast imaging material, along with perfluoro pentane.^[^ ^171^ ^]^ In vivo experiments have demonstrated the selective accumulation of MPmTNs at plaque sites, facilitating macrophage apoptosis and disruption of activated platelets within the plaques.

## 6 CONCLUSIONS AND PERSPECTIVES

The enhanced comprehension of the pathophysiological mechanisms underlying atherosclerosis, combined with the rapid advancements in nanomedicine, has greatly propelled the exploration of drug delivery strategies based on nanotechnology. As most nano‐drugs rely on the enhanced permeability and retention effect to target plaque lesions passively,^[^ ^172^ ^]^ high atherosclerotic blood flow results in poor retention of nano‐drugs at the lesion site,^[^ ^6^ ^]^ resulting in low targeting efficiency and increased non‐selective enrichment, often failing to achieve the desired drug delivery effect. Therefore, more and more researchers are dedicated to exploiting the physiological properties of overexpressed receptors or cytokines at the lesion site by modifying antibodies, targeting peptides, and naturally occurring ligand analogs on the surface of nanoparticles to target specific cytokines and cell surface receptors, thereby increasing the accumulation of drugs at the lesion site and allowing them to exert local therapeutic effects to reduce systemic side effects and improve therapeutic efficacy.^[^ ^173^ ^]^ This paper reviews ligand modification design strategies for nanoparticles targeting atherosclerotic therapeutic aspects over the last five years. These nanoparticles have shown excellent preclinical results and effectively reduced atherosclerotic lesion plaques. However, the application of nanomaterials in reaching clinical atherosclerotic plaque therapy still presents many challenges and pressing issues that need to be addressed.

### 6.1 Avoidance of biological barriers

When NPs circulate in the bloodstream, they encounter complex biological barriers that hinder their efficacy in atherosclerosis treatment.^[^ ^174^ ^]^

#### 6.1.1 Protein corona

One significant challenge is the formation of a protein corona due to protein adsorption, potentially disrupting their targeting capacity and biodistribution. This corona can mask targeting molecules on nano‐drugs, diminishing their binding capability to target cells or tissues and limiting drug distribution.^[^ ^175^ ^]^ Protein adsorption is minimized by functionalizing the NP surface with various polymers such as dextran, polyvinylpyrrolidone, or polyethylene glycol.^[^ ^175^ ^]^ This strategy increases the cyclic half‐life of NPs, but modification with hydrophilic stealth polymers still interferes with targeted functionality.

#### 6.1.2 Immunological clearance

In circulation, nanomedicines may be recognized as foreign bodies by the monocyte–macrophage system (MPS) and phagocytosed, resulting in rapid clearance from the body. This would reduce the concentration of nanodrugs in the blood and limit their accumulation in the target region. Nanoparticles with diameters exceeding the range of 5∼150 nm and a positive charge are susceptible to renal filtration or non‐specific entrapment by liver MPS.^[^ ^176^ ^]^ Notably, although new nanoparticle‐based targeting strategies improve targeting, most administered nanoparticles are difficult to avoid being absorbed by the liver or lungs, or being recognized and cleared by innate immunity.^[^ ^177^ ^]^ Hence, it is crucial to fine‐tune the particle size, morphology, and surface charge to prevent the clearance of NPs. Additionally, studies have revealed that incorporating modifications such as PEG^[^ ^178^ ^]^ or HA^[^ ^179^ ^]^ can effectively reduce the liver uptake of nanoparticles. Furthermore, it is crucial to thoroughly evaluate the variances in cell surface molecule expression across different organs to prevent off‐target effects while designing nanoparticles for atherosclerosis targeting. In specific, the innate immune system's resistance needs to be carefully studied before clinical trials can be conducted.^[^ ^180^ ^]^ Encapsulating nanoparticles with biological membranes (e.g. erythrocyte membranes,^[^ ^181^ ^]^ macrophage membranes,^[^ ^182^ ^]^ platelet membranes,^[^ ^183^ ^]^ and leukocyte membranes^[^ ^184^ ^]^) and using bionanoparticles (e.g. HDL^[^ ^185^ ^]^ and exosomes^[^ ^186^ ^]^) can serve as a beneficial design strategy, as it helps evade rapid clearance by the immune system and mitigates the occurrence of unintended off‐target effects.^[^ ^187^ ^]^

### 6.2 Reducing off‐targeting and improving targeting efficiency

#### 6.2.1 Optimal ligand density

The density of ligands on nanoparticle surfaces is a crucial factor in achieving effective targeting. Recent insights suggest that intermediate ligand densities exhibit superior cellular binding compared to excessively high or low densities.^[^ ^188^ ^]^ This balanced approach enhances the interaction between nanoparticles and target cells, enhancing the overall targeting efficiency. Factors like the unique diffusivity of ligand–receptor pairs and the affinity constant are pivotal in determining the final quantity and uniformity of adsorbed particle surface density.^[^ ^189^ ^]^ Identifying the optimal ratio to optimize targeting efficiency is also a promising avenue for future investigation.

#### 6.2.2 Strategic ligand selection

Selecting appropriate targeting ligands is a pivotal determinant of successful targeting. A comparative study assessed the targeting efficiency of cRGD peptide and Col‐IV, both attached to Pluronic‐loaded iron oxide nanoparticle (IONP), for atherosclerotic plaque targeting in ApoE^−/−^ mice.^[^ ^152^ ^]^ Nanoparticle carriers of comparable size, surface charge, and IONP loading content, yet featuring distinct targeting ligands, underwent assessment through in vitro and in vivo experiments. The outcomes revealed superior efficacy in cRGD‐based targeting over Col IV‐tg peptides. However, there remains a need for deeper exploration into how different ligands influence nanoparticle delivery efficiency. These disparities in targeting strategies impose more rigorous demands for the continued refinement of nanoparticle design.

#### 6.2.3 Embracing multi‐ligand strategies

Current research has focused more on single ligand modification targeting therapies, which rely heavily on the binding affinity of individual ligands to their receptors. Through the continued exploration of nanoparticle synthesis methods, nanoplatforms for multi‐targeted synergistic therapies are constructed, acting on different anti‐atherosclerotic pathways or combining drugs with different functions to optimize the overall therapeutic effect.^[^ ^190^ ^]^ Research is also needed to further elucidate the intracellular signalling pathways at the lesion site, which will contribute to the identification and screening of novel molecular markers, enabling more precise targeting of the lesions.

### 6.3 Challenges in clinical translation

Despite encouraging findings in preclinical research, numerous challenges emerged in the development of nanomedicines targeting atherosclerosis, hindering their progress in clinical translation.

#### 6.3.1 Limitations of preclinical models

Current atherosclerosis models lack full resemblance to human physiology and disease progression.^[^ ^191^ ^]^ While preclinical studies show promise, translating the effectiveness of therapies from animal models to humans is not straightforward. Genetic modifications in animal models may not fully capture human physiological conditions.^[^ ^192^ ^]^ For example, the widely used ApoE^−/−^ mouse model^[^ ^193^ ^]^ does not mirror the polygenic and cholesterol‐dependent nature of human atherosclerosis. These differences necessitate novel preclinical models that better simulate the complex human condition for accurate assessment.

#### 6.3.2 Barriers to commercialization

Despite promising advancements, nanomedicines are not yet ready for widespread commercialization.^[^ ^194^ ^]^ From one point of view, the behaviour of nanosystems in dynamic physiological and pathological changes in vivo is not yet clear and there is a need for a detailed assessment of the pharmacokinetic characteristics, biocompatibility, and safety of nanotherapeutic drugs in preclinical studies. Moreover, most nanomedicines currently designed are administered intravenously and are restricted to in‐hospital administration. However, atherosclerosis represents a chronic condition, necessitating a protracted and comprehensive treatment approach, and this route of administration limits its applicability to the treatment of chronic diseases. Therefore, there is a need for research into dosage forms other than injection, such as nanoformulations for oral^[^ ^195^ ^]^ and nebulized inhalation routes^[^ ^196^ ^]^ of administration. More importantly, commercial nanomedicines need to be able to achieve large‐scale production of nanoparticles under good manufacturing practices, cost‐effective preparation methods, and guaranteed batch‐to‐batch reproducibility, and reliability.^[^ ^197^ ^]^ To achieve these goals, there is a need for increased collaboration between laboratories and pharmaceutical companies to accelerate drug development.

To sum up, although nanomedicine is still in its infancy,^[^ ^198^ ^]^ considering the notable accomplishments of nanomedical methodologies in preclinical explorations of atherosclerosis, the utilization of nanotechnology‐driven targeted therapy offers promising prospects for customized and precise treatment of atherosclerotic plaques. Progressing in this domain necessitates strategic formulation and quantitative assessment tools for understanding how ligands and targets interact, standardizing preclinical variables, and creating holistic technology designs that encompass translational considerations and knowledge dissemination, including the sharing of negative outcomes. We anticipate that these elements will continue to be recognized and tackled collaboratively by academic, industrial, and regulatory communities. We envision that moving forward, a greater number of highly specific, non‐immunogenic, and compact ligands, such as peptides and peptidomimetics, will be harnessed to transform them into nanocarriers, resulting in the creation of more discerning and effective nano‐drug delivery systems.

## CONFLICT OF INTEREST STATEMENT

The authors declare no conflicts of interest.
